# Everyday Out‐of‐Home Activities and Healthy Ageing: A Diary‐Based Analysis of Activity Locations, Purposes and Characteristics and Their Relevance to Environmental Design for Ageing‐in‐Place

**DOI:** 10.1155/jare/3428651

**Published:** 2026-10-19

**Authors:** Ki Tong, Caroline Pearce, Andrew Kingston, Catharine Ward Thompson

**Affiliations:** ^1^ Advanced Care Research Centre University of Edinburgh Usher Building The University of Edinburgh 5-7 Little France Road Edinburgh BioQuarter - Gate 3 Edinburgh EH16 4UX UK ed.ac.uk; ^2^ Population Health Sciences Institute (PHSI) Faculty of Medical Science Newcastle University, Health Innovation Neighbourhood Rm 107 Biogerontology Building Newcastle Upon Tyne NE4 5PL UK; ^3^ OPENspace Edinburgh College of Art Edinburgh School of Architecture and Landscape Architecture 74 Lauriston Place Edinburgh EH3 9DF UK

**Keywords:** ageing-in-place, environmental design, older people, physical activity, social health

## Abstract

**Background:**

Maintaining diverse and outdoor activities is crucial for healthy ageing, yet activity types, environmental settings and health implications have rarely been examined together. Research has characterised activity diversity across cognitive, social and physical domains and a parallel literature has examined the environments in which activity takes place, but activities are typically analysed in isolation from one another and from their settings. What remains less well established is the joint account: work that characterises a range of activity domains as content, locates each in its actual environmental setting and relates that coupling to multidimensional wellbeing. This study takes the out‐of‐home (OOH) trip as its unit of analysis, since it is the smallest unit that carries an activity, a setting and a duration together, and since the settings of OOH activity are those that neighbourhood design, greenspace provision and transport policy are able to shape. It explores the associations between diverse OOH activities, their environmental contexts and health and well‐being outcomes in older adults.

**Methods:**

Twenty‐five community‐dwelling adults aged 50 and over completed a paper activity diary recording all trips outside the home over seven consecutive days. Diaries were completed at up to four time points between winter 2022 and summer 2023, yielding 54 diaries and 742 recorded trips. The diary captured the purpose, setting, distance from home and time spent walking, in a car, on public transport and sitting for each trip, and these were examined in relation to self‐reported health and well‐being measures.

**Results:**

Analysis of 742 trips recorded across 54 weekly diary entries showed that trips for utilitarian and social purposes were the most common, but natural environments were the most frequent location. Recreation trips involved the longest walking times, while social engagement involved the shortest. A higher frequency of recreation and exercise trips was linked to better physical function and quality of life; social activities were associated with better perceived health.

**Conclusion:**

Social engagement appears to be a critical pathway to health, even if sedentary. Nonetheless, natural spaces are chosen environments for a range of OOH activities. We recommend neighbourhoods be designed with multifunctional natural spaces to encourage mobility during OOH trips, supporting ageing‐in‐place.

## 1. Introduction

Policymakers and researchers alike are increasingly focused on enabling individuals to age well in place, which refers to the ability to live safely and independently in one’s own home for as long as possible [1–4]. Enabling people to age in place relies on multiple factors, including the home environment, community resources and support for physical and social activities [5–7].

The ability to perform daily activities is consistently highlighted as a crucial influence on maintaining health and wellbeing in older age [8, 9]. For instance, Havighurst’s [10] activity theory suggests that older adults achieve satisfaction and happiness by sustaining the activities of their middle years into older age, or finding suitable alternatives when some activities must cease. Similarly, Rowe and Kahn [11] argued that healthy ageing encompasses not just freedom from disease or maintained physical and cognitive function, but also active participation in social and productive pursuits. The World Health Organisation (WHO) also strongly emphasises daily activities in promoting healthy and active ageing [12]. The WHO’s healthy ageing model [13] underscores the importance of enabling older adults to maintain mobility, foster relationships and contribute to society, viewing these as reflections of functional ability and the environment and integral to healthy ageing. Therefore, how older adults spend their time in daily activities can serve as a key indicator of their progression into healthy and active ageing [14–17].

Evidence supports the idea of a positive link between spending time out of the home with improved cognitive function, reduced depression and mortality rates among older adults [15, 18, 19, 21]. Increased time spent out‐of‐home (OOH) has been correlated with increased life satisfaction [22–24], reduced psychological stress [25], higher quality of life [26], enhanced psychological wellbeing [27], more positive affect [28] and less negative affect [29, 30]. These positive outcomes could be explained by considering the kind of activities people engage in when they go out and the resulting impact on their overall health status and wellbeing.

The activities that take older adults out of the home have been studied extensively, and a substantial body of work characterises activity diversity across cognitive, social and physical domains [31–34]. A parallel literature examines the environments in which activity occurs, including walkability, residential density, street connectivity, land use mix and destination diversity, access to facilities, pedestrian infrastructure, greenery, lighting and safety [35, 36], alongside work relating built environment characteristics to physical function [37]. Research on activity spaces has further established where activities take place and how far people range in the course of them [38, 39].

This literature has tended to develop in parallel. Activity domains are frequently analysed in isolation from one another, and the settings in which activities occur are more often treated as an antecedent of activity in general than as a property of particular activities. Where diversity across domains has been characterised directly, as in work relating the variety of daily activities to wellbeing and cognitive function [34], the settings in which those activities occur are generally not recorded. What remains less well established is the joint account: work that characterises a range of activity domains as content, locates each in its actual environmental setting and relates that activity and environment coupling to multidimensional wellbeing. A wider range of activities and their corresponding impacts on health might usefully be explored on this basis [40].

Addressing this requires a unit of observation that holds content and context together, and the OOH trip serves this purpose. A trip carries a purpose, a destination, a distance from home and a duration in a single record, so the activity and the setting in which it was performed are coupled at the point of collection rather than joined afterwards through aggregate measures of the residential neighbourhood [39]. Recording trips as they occur also captures settings people travel to rather than only those they live among, which matters where activity ranges beyond the immediate neighbourhood. The focus additionally locates the analysis where environmental intervention is possible, since the places in which OOH activity is performed are those that neighbourhood design, greenspace provision and transport policy can act upon, whereas activity within the home lies largely beyond their reach.

Existing research connects physical movement and outdoor activities to wellbeing [38, 41, 42], but the full picture remains incomplete, particularly regarding everyday mobility. Mobility is not merely physical travel from point A to B; it is an embodied practice intrinsically tied to a person’s sense of freedom and autonomy [43, 44]. Everyday mobility emphasises individual movements through space, defined as ‘short distance, high frequency’ movements, whether that is through walking, driving, use of public transport, cycling, or other means [45]. This concept refers to how much people get ‘out and about’ and is related to health and quality of life through a number of pathways [46]. One pathway is through the physical health benefits derived from modes like walking [45], which are affected by both person‐related factors and the outdoor environment [47, 48]. Other pathways might also link mobility to quality of life without requiring physical activity as the mechanism. For instance, activities of daily living, such as trips to the shops or to visit friends, maintain the independence prized by many as a ‘building block’ for quality of life at an older age [49]. By employing activity diaries to focus on OOH trips, the current study moves beyond simply measuring physical activity and explores the role of this everyday mobility in maintaining independence and contributing to flourishing in later life.

Where an activity takes place matters not only for the character of the setting but also for how far it sits from home, and life space offers an established way of capturing this. Life space refers to the spatial extent within which a person moves over a given period, arising from the interaction between a person’s capabilities and the demands of the environment they encounter [50]. It has been associated with social participation, quality of life and physical activity in later life, and is widely used as a self‐report measure of community mobility [51, 52]. Life space is not employed here as a general account of environment. It describes spatial reach, indicating how far a person’s activities extend beyond the home, and is used alongside the coding of activity locations, which describes the character of the settings themselves. Together these give the two properties of setting that this study requires: where an activity happened and how far the person travelled to reach it.

While advancements in technology, such as smartphones, are increasingly used to assess mobility, diary methods remain a valuable and widely used tool for capturing activity patterns and time spent OOH [53–55]. This is particularly relevant given that smartphone ownership remains lower among older adults than among younger populations, a gap that has narrowed but not closed over the past decade [56, 57]. Recent figures in the US indicate that 78% of adults aged 65 and over own a smartphone, compared with 97% of those under 50% and 90% of those aged 50 to 64 [57], while in the UK 13% of people aged 65 and over and 20% of those aged 75 and over have no home internet access [58]. Time‐use diaries are considered more precise and accurate than survey questions for understanding daily lives, as they ask participants to record activities in their usual context and allow participants to reflect on what they have been doing and why [59, 60]. However, they have been criticised for relying on retrospective recall, which may limit certain aspects of validity and reliability.

This paper aims to investigate the multidimensional nature of older adults’ OOH activity, their environmental settings, and their relationship with self‐report health and wellbeing. The study employed a diary‐based methodology to capture the details of outdoor trips—defined as instances of leaving or going outside the home—and examine whether the purpose, distance and time spent on these OOH activities are associated with self‐reported health and functional status.

Brian Little’s Personal Project Methodology (PPM) [61] offers a person‐centred framework for exploring the knowledge gaps detailed above and corresponding association with wellbeing. PPM considers a wide range of pursuits as projects where activities are “extended sets of personally salient actions in context” [62]. The approach captures self‐report meaning an individual assigns to their goals, emphasising the social and physical contexts that facilitate or hinder these projects [63, 64]. While environmental factors are acknowledged for their association with health, the connection between these factors and older people’s individual goals is less well understood [65]. Its established use in research makes PPM ideal for categorising and understanding the self‐report meaning of older adults’ OOH activities.

### 1.1. Current Study and Research Questions

Based on the identified gaps concerning the types and patterns of activities and the need for a more granular examination of daily mobility, the current study aims to investigate the complex landscape of older adults’ OOH activities. For the purposes of this paper, we consider ‘activities’ in a broad sense as any pursuits that individuals engage in, encompassing physical activity, social engagement, leisure and productive pursuits. We define ‘OOH activities’ as those carried out beyond the indoor home environment, including but not limited to engagement with nature (e.g., gardening and visiting parks), social participation (e.g., meeting friends and attending community events), leisure and recreation (e.g., walking, cycling and sports) and productive pursuits (e.g., shopping, volunteering and caregiving or paid work conducted outside the home). Travel mode is treated as an attribute of a trip rather than as a category of activity. Drawing on the literature on everyday mobility, independence and functional ability [45, 49], we posit that simply getting out of the house for any kind of activity might be an important indicator of ageing well. The scope is not restricted to habitual or routine activity. The diary recorded every trip taken within a 7‐day window, whether recurrent or exceptional, and no distinction was drawn between the two at the point of collection or in coding. ‘Everyday’ is therefore used to denote activity as it occurs across an ordinary week, rather than to designate a subset of activities that recur.

The diary recorded time spent walking, in a car, on public transport and sitting for each trip, and all four are examined at the person level in relation to health and wellbeing. Walking time is used as the trip‐level metric because it is the component of a trip most directly shaped by the design and management of the environments through which people move and because it is repeatedly shown to be associated with active participation and physical health in older age [47, 48]. Time in a car or on public transport reflects route length, traffic conditions and service provision as much as the traveller’s own engagement. This focus is analytic rather than evaluative, and we return in the discussion to what a fuller treatment of motorised modes would require. This research specifically addresses the following questions:

• RQ1: What types of everyday activities do older adults engage in on OOH trips based on their main purposes, that is, the self‐reported reason for the trip, coded into 10 categories and amalgamated into four higher‐level groupings (engagement with nature, engagement with people, recreation and exercise and utilitarian), and where they took place?
• RQ2 How do walking time and time spent walking, as the component of a trip most directly shaped by the environments through which people move, differ between different characteristics of the everyday OOH activity trips in terms of their main purposes, distance from home and locations where they took place?
• RQ3: How do the identified everyday outdoor activity types and patterns (including time spent and frequency) relate to older adults’ physical health and wellbeing?

## 2. Methods

### 2.1. Activity Diaries

This study employed diary methods to explore the complex landscape of older adults’ OOH activities. Diary methods have a long history in research with older adults to gather information on time use [66], daily activities [67–69], experiences of chronic health conditions [70, 71] and travel and mobility patterns [42, 72–75]. Depending on the study’s focus, the diary format can vary, allowing researchers to gather both quantitative and qualitative data over different durations [76].

Time‐use diaries are particularly useful for capturing the trajectory of daily activities over a set period, such as a day or a week [77]. Research with older adults has shown that this method can deepen the understanding of the temporal organisation of daily life, providing insight into where and how activities are sequenced together [68, 78]. Furthermore, time‐use diaries can shed light on the choices people make regarding their day‐to‐day activities and how these activities contribute to their meaningful life context [66, 68, 71].

### 2.2. Study Context

This paper presents findings from activity diaries completed by older adults as part of a larger, longitudinal, mixed‐methods study that investigated the role of the local outdoor environment in supporting older adults in Scotland, UK, to pursue ‘personal projects’ and the impact on their health and quality of life [79]. The study is part of a wider cohort of studies hosted within the Research Centre.

Participants were recruited from five areas in Scotland. Study areas included a mix of socio‐economic contexts based on the Scottish Index of Multiple Deprivation (SIMD), acknowledging both the lack of representation of people living in deprived areas in studies of environment and health and the social inequalities that impact the experience of healthy ageing [80]. Study areas were chosen from the Central Belt of Scotland, the most urbanised part of the country, where approximately 70% of the population lives, to encompass diverse urban and semirural settings in the east of Scotland.

### 2.3. Recruitment

A total of 25 participants took part in the current study, with demographic information presented in Table 1. Older adults were defined as individuals aged 50 and over to account for the differences in life expectancy within Scotland, particularly in more deprived areas. Participants were recruited primarily via local community organisations within the selected study areas, including visiting community centres and participating in events such as walking groups and coffee mornings attended by older adults. After receiving an invitation to attend, the second author visited the groups to speak with members about the research and to disseminate project flyers and information sheets. Two participants were recruited online through social media groups. Consideration was also given to the inclusion of various housing types and a mix of urban and semirural areas. Participants were given a £15 retail voucher as a token of appreciation for their contribution.

**TABLE 1:** Demographic information of participants.

	Age				Total
	50–59	60–69	70–79	80–89	
Count	2	7	9	7	25
% of total	8.00%	28.00%	36.00%	28.00%	100.00%

	**SIMD**				
	**1–4**	**5–7**	**8–10**		**Total**

Count	12	7	6		25
% of Total	48.00%	28.00%	24.00%		100.00%

*Note:* SIMD is a relative measure of area‐based deprivation across Scotland, calculated by combining seven domains: income, employment, education, health, access to services, housing and crime. It ranks small areas (data zones) from most deprived (rank 1) to least deprived (rank 10) [105].

### 2.4. Procedure

Ethical approval for the study was provided by the University Ethics Committee. All study materials, including participant information sheets, consent forms and interview schedules, were reviewed by public contributors from the Patient and Public Involvement network to ensure their appropriateness for older participants. Prior to participation, those interested were provided with a Participant Information Sheet and asked to sign a consent form.

In the larger study, all participants took part in a semistructured interview, which collected socio‐demographics, health, mobility, and quality of life measures. These interviews took place at three time points: winter 2022, spring/summer 2022 and spring/summer 2023. Following the interviews, participants were invited to complete a weekly activity diary, documenting activities outside their home for seven consecutive days. Participants were invited to complete a weekly activity diary across four time points: winter 2022, spring/summer 2022, winter 2023 and spring/summer 2023. One diary was returned at the first point, followed by 16, 21 and 16 at the three subsequent points, giving 54 diaries in total.

The number of diaries completed varied between participants for two main reasons. Recruitment continued throughout the study period, so participants entered at different points, and those joining later could not contribute to earlier collection periods. Recruitment also began while restrictions associated with the COVID‐19 pandemic were still in place, which limited the number of participants enrolled at the first collection period and accounts for the single diary returned then. A smaller number of participants did not complete a diary at a subsequent time point, whether through nonresponse to follow‐up or withdrawal from the study.

Printed activity diaries were sent to participants by post with instructions for their completion, along with a stamped addressed envelope for their return. Participants were asked to complete the diary over the course of 1 week, choosing any day they wished to start. They were instructed to fill out the diary at the end of each day.

### 2.5. Materials and Measures

Participants were asked to complete an activity diary for seven consecutive days to record all times they went outside their home. The diaries allowed participants to record up to four trips involving OOH activities a day. The diary was a printed A4 booklet with colour‐coded sections for each day and was based on activity diaries used in a previous study [81]. For each OOH trip, participants were asked to record the time of day they left and returned home; whether they were alone or with someone; the main purpose of going out; the location they went to and distance from home; and time spent in different modes of travel, including walking, in a car and in public transport, as well as the time spent sitting and any other activities they did while they were out (see Supporting Information Appendix A for the detailed format of the diary). Distance from home was based on four of the six life‐space mobility levels derived from the Life‐Space Assessment (LSA) [51, 52], which measures a person’s ability to move through different spatial areas, including outside the home, within the local neighbourhood, outside the local neighbourhood but within town and outside town. Wellbeing outcomes were assessed using standardised, validated scales during semistructured interviews conducted at winter 2022, spring/summer 2022 and spring/summer 2023 of the larger study. Health‐related quality of life was measured using the EURO‐QOL 5D‐3L (EQ‐5D‐3L), and functional status was measured using an Instrumental Activities of Daily Living (IADL) scale, adapted from Jette [82]. Participants also self‐rated their health using a five‐point scale and the EQ‐visual analogue scale (VAS). Demographic information collected in the questionnaire included age, sex, ethnicity, living arrangement (alone or with someone), educational attainment and former or current occupation (of the household’s main earner).

### 2.6. Data Analysis

To identify the types of activities, the free‐text responses from the diaries were coded independently by the first and second authors. Following previous research using PPA [83], the main purpose of going out for each OOH trip recorded by participants was coded into 10 categories; the relevant categories were then amalgamated into four higher‐level categories: engagement with people, recreation and exercise, engagement with nature, and utilitarian (Table 2). These were used to classify types of activity undertaken. Location was also coded deductively based on 10 place codes generated through analysis of the data collected [79] as part of the wider study and categorisations based on previous research using PPA [83]. Numerical codes were assigned to trip types and places according to the primary purpose of the trip and where the activity took place, and these were input into a spreadsheet. The spreadsheet also contained demographic and health and quality of life measures. Summer 2022 quality of life data were used for the winter and summer 2022 diaries, and summer 2023 quality of life data were used for the winter and summer 2023 diaries.

**TABLE 2:** High‐level categories for the main purpose of going for each OOH trip.

High‐level categories	Main purpose of going out on OOH trip	
	Categories	Examples
Engagement with nature	Gardening	e.g., plant maintenance
	Relaxation‐related activities	e.g., reading and listening to music
	Walking in nature	e.g., a stroll in an urban park
	Other engagement with nature	e.g., bird‐watching

Engagement with people	Participation in meetings	e.g., attending events at community centres
	Meeting people	e.g., coffee with friends

Recreation and exercise	Doing sports	e.g., lawn bowling
	Trips for leisure activities	e.g., museum
	Walking (not in nature)	e.g., walking to town

Utilitarian	Utilitarian trips	e.g., grocery and worship

For RQ3, data were aggregated to the participant level to create a single record for each individual. For the trip‐based metrics, data from all completed diary entries were combined. This involved calculating overall averages (e.g., total weekly walking time divided by total number of trips) and total counts (e.g., number of trips per week), and total sums (e.g., weekly walking time) for trip‐based metrics. Physical health (self‐reported health and IADL) and well‐being scores were measured at each interview of the larger study, and the average score for each participant was calculated across these waves. This method was adopted specifically to address the varying number of diary entries completed by participants and potential variation over time or season in health and wellbeing outcomes, ensuring that each individual contributed a single, person‐level data set for understanding the association with the participant’s overall health and wellbeing. No missing scores were observed in the sample.

Statistical analysis was performed in R. We first conducted descriptive statistics to summarise the data. The unit of analysis was based on each individual trip (defined as a round trip from home). Since the details associated with each trip were unique, the total dataset included a variable number of entries per participant based on their engagement with the diary method. Nonparametric modelling was used for the primary analyses due to the distribution and nature of the data. The data have a nested structure, with multiple trips recorded per participant. Nonparametric methods address the distributional properties of the data but do not remove the dependency created by repeated observations within individuals. The small number of participants and the uneven number of diaries completed would make multilevel estimates unstable, so a sensitivity analysis was conducted instead. One trip per participant was drawn at random, and the trip‐level analyses were rerun on the resulting independent dataset of 25 trips. This was repeated 1000 times, and results are summarised across repetitions rather than pooled, as a check on whether the findings are driven by within‐participant clustering.

## 3. Results

### 3.1. RQ1: What Types of Activities Do Older Adults Engage in OOH Trips Based on Their Main Purposes and Where They Took Place?

Twenty‐five participants completed 54 diaries in total, yielding 742 recorded trips. Eleven participants completed a diary at three time points, seven at two and seven at one. Participants completing one, two and three diaries differed on health and functional characteristics. Median IADL scores were 24.0, 15.0 and 13.5, respectively, indicating greater difficulty with instrumental activities among those contributing fewer diaries, with median EQ‐5D‐3L scores of 0.443, 0.691 and 0.796 and median perceived health scores of 60.0, 80.0 and 82.5. Diary behaviour was comparable, with median outdoor time per trip of 128.8, 139.8 and 139.1 min. Because recruitment continued throughout the study, the number of diaries completed reflects when a participant entered as well as their subsequent participation, so these groups differ in composition as well as in contribution. The consequence for the analysis is that trips are unevenly distributed across participants, and this is addressed directly by the sensitivity analysis described below, in which each participant contributes a single trip irrespective of the number of diaries completed.

To quantify how common specific types of activity were across different participants, a participant‐level prevalence measure was constructed: for each of the categories coded for the main purpose of going out, an individual was counted once if they identified that category at least once in any time point of measurement, irrespective of the number of trips recorded in that category. This procedure prevents inflation of counts by repeated entries from the same individual.

Utilitarian trips and meeting people showed the highest prevalence, followed by trips for leisure activities, walking in nature, walking (not in nature), and gardening. Participating in meetings, doing sports, relaxation‐related activities, and other engagement with nature were less prevalent. Figure 1 presents participant‐level prevalence by category.

**FIGURE 1:**
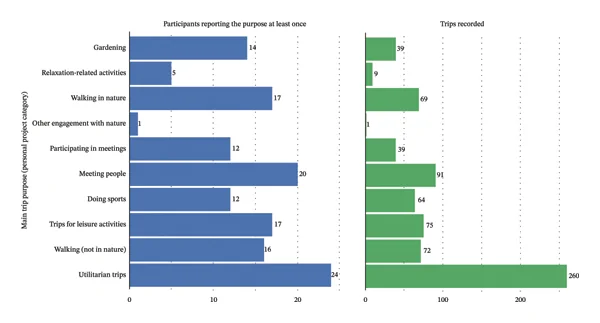
Number of participants whose reported at least once and number of trips for each main trip purpose.

The frequency of activities for different purposes was calculated by counting the number of trips assigned to each purpose category in Table 2 across all waves and participants. Utilitarian trips were the most frequent—and the most consistent—category across waves, followed by meeting people, trips for leisure activities, walking (not in nature), and walking in nature. Relaxation‐related activities and other engagement with nature were comparatively infrequent. Figure 1 displays trip‐level purpose category counts.

The most commonly reported location where activities occurred was determined by calculating the counts of the locations where the main purpose of the trip took place, based on previous research using PPA [83]. Natural environments were the most frequently reported, despite nature‐related activities not being the most common purpose for trips overall (Figure 2). Within natural environments, greenspace was the predominant subcategory, exceeding bluespace, combined green–blue spaces, and private gardens/allotments (Figure 3).

**FIGURE 2:**
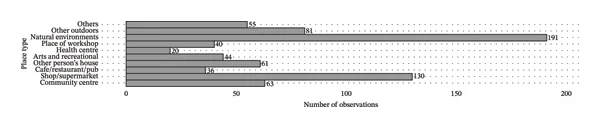
Frequency of locations by main trip purpose.

**FIGURE 3:**
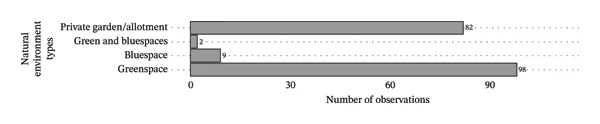
Frequency of natural environment types.

Most trips were undertaken within the local neighbourhood or outside the local neighbourhood but within town. Among the more common activity types, utilitarian trips and meeting people most frequently occurred outside the local neighbourhood, followed by within the local neighbourhood. Walking (not in nature) was more often undertaken within the local neighbourhood than outside it, whereas for walking in nature the counts were comparable across these two distances. Across activity categories, counts at the extremes of the distance scale—outside the home and outside town—were consistently low. Figure 4 displays counts by activity and distance; Figure 5 shows the number of individuals engaging in each activity by distance.

**FIGURE 4:**
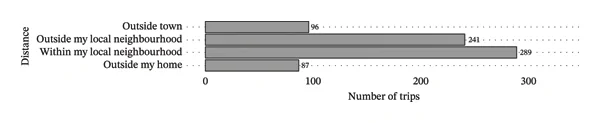
Count of trips based on distance.

**FIGURE 5:**
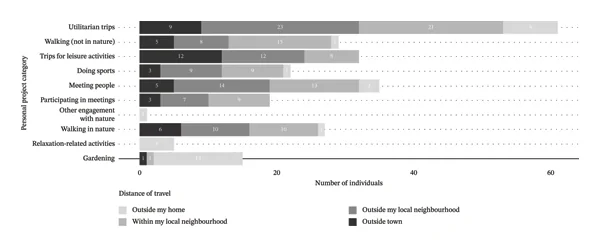
Count of individuals engaged in different main trip purposes based on personal project categories at different distance.

### 3.2. RQ2: How Does Walking Time Differ Between Different Characteristics of the OOH Activity Trips in Terms of Their Main Purposes, Distance From Home and Locations Where They Took Place?

The average weekly walking time per person was 460.08 min (SD = 338.87; range = 0–1515). For trips involving walking, the average walking time per trip was 51.23 min (SD = 52.65; range = 0–360), comprising 0.50 (50%) (SD = 0.35 [35%]) of the overall OOH time for those trips.

For trips involving walking, the main purpose of the trip for the majority was related to either recreational and exercise activities or utilitarian purposes (Figure 6). A Kruskal–Wallis test was conducted to determine if walking time differed significantly across the main trip purposes. Mean rank measures, used in nonparametric tests, reflect the average ordering of scores, where a higher mean rank indicates a tendency toward longer walking time. The results indicated a significant difference in walking time across the activity types, *H*_(3)_ = 55.38, *p* < 0.001, *ϵ*2 = 0.11.

**FIGURE 6:**
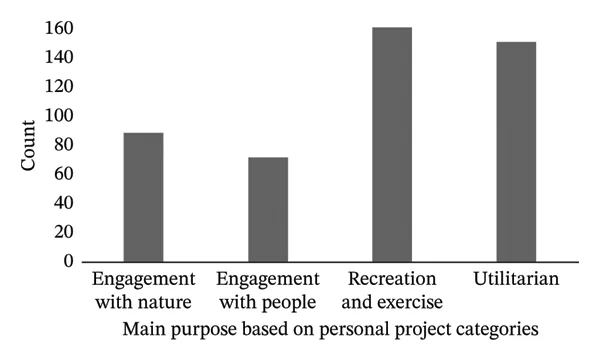
Count of walking trips based on main purpose of trips.

The mean ranks for walking time, ordered from highest to lowest, were: engagement with nature (289), recreation and exercise (273), utilitarian (205) and engagement with people (160). Post hoc comparisons using the Holm‐adjusted Dunn test revealed several significant differences. Specifically, utilitarian trips had significantly shorter walking times than those for engagement with nature (*Z* = −4.62, *p* < 0.000) and recreation and exercise (*Z* = −4.40, *p* < 0.000). Conversely, utilitarian trips had significantly longer walking times than trips for engagement with people (*Z* = 2.30, *p* = 0.043). Further analysis indicated that engagement with people had significantly shorter walking times compared to engagement with nature (*Z* = −5.97, *p* < 0.000) and recreation and exercise (*Z* = 5.83, *p* < 0.000). All other comparisons were not statistically significant. This suggests that trips with the primary aim of engaging with people consistently involved less walking time than all other trip purposes. Table 3 provides a summary of mean ranks and the difference in walking time.

**TABLE 3:** Summary of the Kruskal–Wallis tests on the difference in walking time between the main purposes of trips.

	*n* (mean rank)	Engagement with nature	Engagement with people	Recreation and exercise	Utilitarian
Engagement with nature	89 (289)	—	**−5.970** **p** < 0.001^**∗∗∗**^	−0.906 *p* = 0.365	**−4.621** **p** < 0.001^**∗∗∗**^
Engagement with people	72 (160)	—	—	**5.831** **p** < 0.001^**∗∗∗**^	2.295 *p* = 0.043
Recreation and exercise	161 (273)	—	—	—	**−4.395** **p** < 0.001^**∗∗∗**^
Utilitarian	151 (205)	—	—	—	—

*Note:* Bold values indicate statistically significant results (*p* < 0.05).

^∗^ *p* < 0.05.

^∗∗^ *p* < 0.01.

^∗∗∗^ *p* < 0.001.

A Kruskal–Wallis test was conducted to determine if the distance from home (measured at four levels as elucidated in materials and measures) differed significantly across activity types for trips involving some walking. Mean rank measures, used in nonparametric tests, reflect the average ordering of scores, where a higher mean rank indicates a tendency toward longer distance. The results indicated a significant difference in distance across activity types, *H*_(3)_ = 73.79, *p* < 0.001, *ϵ2* = 0.10.

The mean ranks for distance, ordered from highest to lowest, were: recreation and exercise (409), engagement with people (373), utilitarian (363) and engagement with nature (222). Post hoc comparisons using the Holm‐adjusted Dunn test revealed that engagement with nature trips had significantly shorter distances from home than utilitarian trips (*Z* = −6.10, *p* < 0.000), engagement with people (*Z* = −8.41, *p* < 0.000), and recreation and exercise (*Z* = −6.56, *p* < 0.000). Utilitarian trips also had a significantly shorter distance from home than recreation and exercise trips (*Z* = 2.52, *p* = 0.04). All other comparisons were not statistically significant. This suggests that trips for the purpose of engaging with nature were consistently closer to home than all other trip purposes. Table 4 provides a summary of these results.

**TABLE 4:** Summary of the Kruskal–Wallis tests on the difference in distance from home between the main purposes of trips.

	*n* (mean rank)	Engagement with nature	Engagement with people	Recreation and exercise	Utilitarian
Engagement with nature	118 (222)	—	**−6.10** **p** < 0.001^**∗∗∗**^	**−8.41** **p** < 0.001^**∗∗∗**^	**−6.56** **p** < 0.001^**∗∗∗**^
Engagement with people	127 (373)	—	—	−1.66 *p* = 0.190	0.44 *p* = 0.650
Recreation and exercise	211 (409)	—	—	—	**2.52** **p** = 0.040^**∗**^
Utilitarian	253 (363)	—	—	—	—

*Note:* Bold values indicate statistically significant results (*p* < 0.05).

^∗^ *p* < 0.05.

^∗∗^ *p* < 0.01.

^∗∗∗^ *p* < 0.001.

A Kruskal–Wallis test was conducted to determine if walking time differed significantly across various locations where the main purpose of the trip’s activity took place. Mean rank measures, used in nonparametric tests, reflect the average ordering of scores, where a higher mean rank indicates a tendency toward longer walking time. The results indicated a significant difference in walking time across the place types, *H*_(9)_ = 44.05, *p* < 0.001, *ϵ2* = 0.07.

Specific post hoc comparisons using the Holm‐adjusted Dunn test revealed that walking time for trips with main purpose to go to café/restaurant/pub was significantly shorter when compared to activities in arts and recreational venues (*Z* = −3.62, *p* = 0.01), natural environments (*Z* = −4.00, *p* < 0.01) and other outdoor areas (*Z* = −4.27, *p* < 0.01).

A similar pattern was observed for trips where the main purpose was to go to another person’s house. Walking time for these trips was significantly shorter compared to activities in arts and recreational venues (*Z* = −3.83, *p* = 0.011), natural environments (*Z* = −4.13, *p* = 0.011) and other outdoor areas (*Z* = −4.43, *p* = 0.013).

The mean ranks for the places with significant results were: café/restaurant/pub (138), other person’s house (135), natural environments (263) and other outdoors (218). These findings collectively suggest that activities at food and drink establishments and at another person’s house involved significantly shorter walking times compared to those in natural and other outdoor environments. The walking time between food and drink establishments and other people’s houses was not significantly different. Table 5 provides a summary of mean ranks and the difference in walking time between locations.

**TABLE 5:** Summary of Kruskal–Wallis tests on the difference in walking time between trip purpose locations.

Place	*n* (mean ranks)	Community centre	Shop/supermarket	Cafe/restaurant/pub	Other person’s house	Arts and recreational	Health centre	Place of workship	Natural environments	Other outdoors	Others
Community centre	40 (207)	—	−0.51 *p* = 1.000	−1.94 *p* = 1.000	2.26 *p* = 0.760	1.94 *p* = 1.000	0.99 *p* = 1.000	0.23 *p* = 1.000	−1.92 *p* = 1.000	−2.42 *p* = 0.540	−0.14 *p* = 1.000
Shop/supermarket	97 (234)	—	—	−2.68 *p* = 0.280	−3.00 *p* = 0.120	1.79 *p* = 1.000	−1.35 *p* = 1.000	−0.62 *p* = 1.000	1.90 *p* = 1.000	2.46 *p* = 0.500	−0.29 *p* = 1.000
Cafe/restaurant/pub	25 (138)	—	—	—	0.39 *p* = 1.000	**3.62** **p** = 0.0112^**∗**^	−0.40 *p* = 1.000	−1.16 *p* = 1.000	**−4.00** **p** < 0.001^**∗∗∗**^	**−4.27** **p** < 0.001^**∗∗∗**^	−1.96 *p* = 1.000
Other person’s house	20 (135)	—	—	—	—	**3.83** **p** = 0.010^**∗**^	0.69 *p* = 1.000	−1.44 *p* = 1.000	**4.13** **p** = 0.012^**∗**^	**4.43** **p** = 0.013^**∗**^	−2.26 *p* = 0.776
Arts and recreational	33 (276)	—	—	—	—	—	2.27 *p* = 0.800	1.57 *p* = 1.000	0.57 *p* = 1.000	−0.11 *p* = 0.912	1.67 *p* = 1.000
Health centre	11 (167)	—	—	—	—	—	—	−0.56 *p* = 1.000	−2.16 *p* = 0.941	−2.51 *p* = 0.452	−1.06 *p* = 1.000
Place of workshop	10 (201)	—	—	—	—	—	—	—	1.39 *p* = 1.000	1.77 *p* = 1.000	0.32 *p* = 1.000
Natural environments	136 (263)	—	—	—	—	—	—	—	—	−0.92 *p* = 1.000	1.55 *p* = 1.000
Other outdoors	70 (279)	—	—	—	—	—	—	—	—	—	—
Others	31 (218)	—	—	—	—	—	—	—	—	—	—

*Note:* Bold values indicate statistically significant results (*p* < 0.05).

^∗^ *p* < 0.05.

^∗∗^ *p* < 0.01.

^∗∗∗^ *p* < 0.001.

A sensitivity analysis assessed whether the trip‐level findings depend on participants who contributed more trips. One trip per participant was drawn at random, and the comparisons were rerun on the resulting independent dataset, repeated across 1000 replicates, with the benchmark being the 5% of replicates that would reach significance if no association were present.

The difference in walking time by trip purpose reached significance in 15.8% of replicates, with a median *H*_(3)_ = 4.67, and the ordering of purposes was stable: engagement with nature ranked highest for median walking time in 41.4% of replicates and recreation and exercise in 36.0%, while engagement with people ranked lowest in 63.1%. The difference in distance from home by purpose reached significance in 15.2% of replicates, with a median *H*_(3)_ = 4.31, and here the ordering was more stable still, with engagement with nature ranking nearest to home in 76.8% of replicates and recreation and exercise ranking furthest in 41.6%.

Both purpose‐based comparisons therefore reach significance around three times more often than chance would produce and preserve their orderings, indicating that they are not artefacts of within‐participant clustering, although at 25 observations each replicate is considerably less powerful than the full analysis. The comparison of walking time across locations could not be assessed on the same basis, since 25 trips distributed across 10 place categories leave most categories empty in any single replicate. Applying the procedure to a merged set of seven categories yielded significance in 4.7% of replicates, at the rate expected by chance, so the differences in walking time between locations should be read as descriptive of the trips recorded rather than as generalisable associations.

These results indicate that the association between trip purpose and walking time persists when each participant contributes a single observation, although at 25 observations the test is considerably less powerful than the full analysis. The differences by distance from home and by location are not supported under independence and rest substantially on repeated trips from the same participants. They should accordingly be read as descriptive of the recorded trips rather than as generalisable associations.

### 3.3. RQ3: How Do the Identified Outdoor Activity Types and Patterns (Including Time Spent and Frequency) Relate to Older Adults’ Physical Health and Wellbeing?

Spearman’s rank‐order correlations were performed to assess the relationships between aggregated trip‐based metrics and self‐reported health outcomes, specifically VAS scores for perceived health, EQ‐5D‐3L scores for quality of life, and IADL scores for functional abilities. The range of aggregated metrics included averages of time spent using a car, public transportation, sitting, and walking; counts of trips using a car, public transportation, sitting, and walking; the overall number of trips per person; and total time spent using a car, public transportation, sitting, walking, and out‐of‐house.

Our participants’ mean VAS general health score was 80.61 (SD = 11.78) (0 = ‘Worst imaginable health state’; 100 = ‘Best imaginable health state’). The mean IADL score for performing activities of daily living was 16.42 (SD = 7.03) (11 = independent; 55 = fully dependent). Participants’ mean EQ‐5D‐3L score for quality of life was 0.81 (SD = 0.25) (−0.594 = worst possible health‐related quality of life status; 1.0 = best possible health‐related quality of life status).

Physical function (IADL scores) demonstrated a greater number of significant associations with the various aggregated trip metrics than either VAS and EQ‐5D‐3L. A significant negative correlation was observed between IADL scores, the count of car use frequency (rs = −0.45, *p* = 0.033), and total time spent outdoors (rs = −0.53, *p* = 0.011). Given that lower IADL scores indicate better functional abilities, these negative correlations suggest that individuals with better physical function tended to report more frequent car use and greater total time spent out of house. Additionally, the negative correlation between the count of walking trips and IADL scores was marginally significant (rs = −0.40, *p* = 0.070), indicating a tendency for individuals with better functional abilities to engage in more frequent trips involving walking, although this association did not reach conventional statistical significance.

Neither average walking time nor the count of walking trips yielded statistically significant correlations with EQ‐5D‐3L (quality of life) scores or VAS (overall health) scores. Conversely, average sitting time as part of trips outside the home was found to be significantly and positively correlated with both EQ‐5D‐3L scores (rs = 0.48, *p* = 0.035) and VAS scores (rs = 0.48, *p* = 0.034). This indicates that individuals reporting higher quality of life and better health tended to exhibit longer average sitting times for their trips. While positive, the average time spent on sitting did not correlate significantly with the average time spent outdoors and average walking time. Table 6 shows the correlations between the averages of trip metrics. Furthermore, the count of car use showed a marginally significant correlation with EQ‐5D‐3L scores (rs=0.41, *p* = 0.065), suggesting a tendency for individuals with higher quality of life to engage in more frequent car trips. For car use and public transport, the same three aggregated trip‐based metrics were derived as for walking: the average time per trip, the sum of time recorded across diary entries, and the count of trips involving that mode. Each was entered into the Spearman correlations described above. Aside from the associations between the count of car trips and IADL and EQ‐5D‐3L scores noted above, none reached statistical significance.

**TABLE 6:** Spearman’s rank‐order correlations for the averages of trip metrics.

	Average time spent on walking	Average time spent on sitting	Average time spent on driving a car	Average time spent on public transportation	Average time spent out of home
Average time spent on walking	—	rho = −0.017 *p* = 0.929	rho = −0.257 *p* = 0.179	rho = 0.152 *p* = 0.588	**rho = 0.345** **p** = 0.049^**∗**^
Average time spent on sitting	—	—	rho = −0.126 *p* = 0.541	**rho = 0.704** **p** = 0.005^**∗∗**^	rho = 0.085 *p* = 0.660
Average time spent on driving a car	—	—	—	rho = −0.061 *p* = 0.843	rho = −0.066 *p* = 0.735
Average time spent on public transportation	—	—	—	—	rho = 0.127 *p* = 0.652
Average time spent out of home	—	—	—	—	—

*Note:* Bold values indicate statistically significant results (*p* < 0.05).

^∗^ *p* < 0.05.

^∗∗^ *p* < 0.01.

^∗∗∗^ *p* < 0.001.

Regarding the interrelationships among the health outcome measures themselves, physical functional scores (IADL) were negatively correlated with both quality of life (EQ‐5D‐3L) (rs = −0.72, *p* < 0.001) and perceived health (VAS) (rs = −0.56, *p* = 0.006). These findings suggest that individuals with better physical functions generally reported superior quality of life and higher perceived health. Moreover, VAS scores were, as might be expected, significantly and positively correlated with quality of life (EQ‐5D‐3L) (rs = 0.74, *p* < 0.001), indicating that better perceived health is strongly associated with higher quality of life. A summary of the results above is available in Table 7.

**TABLE 7:** Spearman’s rank‐order correlations between trip‐based metrics and health and wellbeing measurements.

	Quality of life (EQ‐5D‐3L)	Visual analogue scale—self‐rated health (VAS)	Physical function (IADL)
Average time spent on using cars	rho:.005 *p* = 0.991	rho = −0.126 *p* = 0.681	rho = −0.044 *p* = 0.922
Average time spent on using public transportation	rho = 0.500 *p* = 0.140	rho = 0.209 *p* = 0.627	rho = −0.427 *p* = 0.245
Average time spent on sitting	**rho = 0.477** **p** = 0.035^**∗**^	**rho = 0.480** **p** = 0.034^**∗**^	rho = −0.406 *p* = 0.088
Average time spent outdoors	rho = 0.179 *p* = 0.521	rho = 0.158 *p* = 0.543	rho = −0.208 *p* = 0.437
Average time spent on walking	rho = 0.005 *p* = 0.991	rho = −0.132 *p* = 0.628	rho = −0.099 *p* = 0.735
Count of trips involving car use	rho = 0.405 *p* = 0.065	rho = 0.352 *p* = 0.118	**rho = −0.454** **p** = 0.033^**∗**^
Count of trips involving public transportation	rho = −0.097 *p* = 0.735	rho = −0.102 *p* = 0.734	rho = −0.081 *p* = 0.787
Count of trips involving sitting	rho = −0.011 *p* = 0.988	rho = −0.197 *p* = 0.468	rho = −0.075 *p* = 0.803
Count of trips involving walking	rho = 0.246 *p* = 0.332	rho = −0.011 *p* = 0.988	rho = −0.397 *p* = 0.070
Sum of trips made	rho = 0.244 *p* = 0.334	rho = 0.098 *p* = 0.735	rho = −0.343 *p* = 0.126
Sum of time spent on using car	rho = 0.294 *p* = 0.222	rho = 0.180 *p* = 0.521	rho = −0.361 *p* = 0.110
Sum of time spent on using public transport	rho = −0.009 *p* = 0.988	rho = −0.174 *p* = 0.523	rho = −0.211 *p* = 0.430
Sum of time spent on using sitting	rho = 0.067 *p* = 0.838	rho = −0.094 *p* = 0.737	rho = −0.182 *p* = 0.516
Sum of time spent out of house	rho = 0.353 *p* = 0.118	rho = 0.199 *p* = 0.466	**rho = −0.525** **p** < 0.011^**∗**^
Sum of time spent on walking	rho = 0.133 *p* = 0.628	rho = −0.168 *p* = 0.528	rho = −0.312 *p* = 0.181
Quality of Life (EQ‐5D‐3L)	—	**rho = 0.740** **p** < 0.000^**∗∗∗**^	**rho = −0.719** **p** < 0.000^**∗∗∗**^
Visual Analogue Scale—Self‐Rated Health (VAS)	—	—	**rho = −0.560** **p** = 0.006^**∗∗**^
Physical Function (IADL)	—	—	—

*Note:* Correlations highlighted in bold are statistically significant. IADL scoring is inverse (lower score = better function). *p* < 0.05^∗^; *p* < 0.01^∗∗^; *p* < 0.001^∗∗∗^.

A series of Spearman’s rank‐order correlations were computed to examine the associations between total aggregated trip metrics, specifically the total counts of different main purposes of trips, and various aspects of health and wellbeing. The raw count for each main trip purpose was aggregated to a total person‐level sum for each participant, and this sum was then divided by the number of entries completed by the individual to account for the varying number of diary entries completed by individuals and used to test for correlation with measurements of health and wellbeing.

While most correlations were not statistically significant, several notable relationships were observed. A positive and significant association was found between the frequency of trips involving recreation and exercise and quality of life (rs = 0.43, *p* = 0.049). This indicates that participants who engaged in a higher count of recreational and exercise‐related trips tended to report a better quality of life. Furthermore, these trips were negatively and significantly correlated with physical function (rs = −0.51, *p* = 0.027). Given that lower scores on the physical function scale indicate better health and independence in mobility, this finding suggests that individuals with more frequent trips for recreation and exercise were more likely to have better physical function.

Another significant finding was the positive correlation between the count of trips involving engagement with people and rated health (VAS) (rs = 0.45, *p* = 0.049). This result indicates that engaging in more frequent trips involving other people is associated with a tendency towards better self‐perceived health, although this correlation was only marginally significant. Table 8 provides a summary of the results.

**TABLE 8:** Spearman’s rank correlation between aggregated main purposes of trips and measurements of health and wellbeing.

	Quality of life (EQ‐5D‐3L)	Visual analogue scale—self‐rated health (VAS)	Physical function (IADL)
Engagement with nature	rho = −0.278 *p* = 0.156	rho = −0.310 *p* = 0.119	rho = 0.314 *p* = 0.119
Engagement with people	rho = 0.319 *p* = 0.119	**rho = 0.448** **p** = 0.049^**∗**^	rho = −0.343 *p* = 0.119
Recreation and exercise	**rho = 0.431** **p** = 0.049^**∗**^	rho = 0.346 *p* = 0.119	**rho = −0.514** **p** = 0.027^**∗**^
Utilitarian	rho = 0.198 *p* = 0.295	rho = −0.106 *p* = 0.555	rho = −0.200 *p* = 0.295

*Note:* Correlations highlighted in bold are statistically significant. IADL scoring is inverse (lower score = better function). *p* < 0.05^∗^; *p* < 0.01^∗∗^; *p* < 0.001^∗∗∗^.

## 4. Discussion

This study examined older adults’ everyday outdoor activities and how trip purposes, places, and distances relate to reported physical function and wellbeing. It is notable that participants in this study were highly active and spent on average at least 7 h per week walking, whereas cross‐sectional analysis based on the Scottish Health Survey suggests that the average for this age group across the population as a whole is only approximately 3 h per week [84]. The results therefore reflect the environmental and behavioural mechanisms of a comparatively healthy and active segment of the older population. Nonetheless, these observations provide information on the environmental and social factors that are associated with relatively higher mobility, which can help point to ways to support broader physical activity and wellbeing across the older adult population as a whole. Where socioecological accounts have related survival and life expectancy to national conditions such as income, health expenditure and infrastructure [85], evidence at this scale speaks to the neighbourhood conditions that policy can act on more directly. While trips where the main purpose was to engage with nature were not the most frequently reported, natural environments were the most frequently reported location for OOH trips. This suggests the natural settings are often a backdrop for OOH activities, even though they are not associated directly with the primary purpose of the trip. This finding supports the findings of Currie et al. [86] that, in a study of barriers and facilitators affecting accessibility to outdoor spaces among older people, natural places were often incidental settings for routine or social activities rather than being destinations themselves. This has implications for how such settings are provided and managed. Since natural environments hosted a wider range of trip purposes than any other location type, their value in this sample lies in accommodating the activities older people already undertake rather than in drawing visits for their own sake. No association was found here between time spent in natural environments and any measure of health or wellbeing, so this is a recommendation about where everyday activity is supported rather than a claim about a measured health benefit of nature exposure.

Trips for the purpose of recreation and exercise were linked to better quality of life and better physical function, but they tended to occur further from home, potentially privileging those with greater mobility or transport access. Socially‐oriented trips involved the least walking time but were associated with better perceived health. Across the sample, most travel occurred within the neighbourhood or within town, emphasising the everyday, near‐home geography of later‐life mobility. These observations support principles of ageing‐in‐place that emphasise the importance of a local physical environment that enables access to a diversity of amenities as conducive to flourishing in later life.

Engagement with nature as the main purpose of the trip involved considerably longer walking time and yet tended to occur closer to home than other key activity types, suggesting that trips undertaken for the purpose of engaging with nature involve more walking than trips undertaken for other purposes. However, this engagement with nature did not correlate with any measures of health or physical function. This runs counter to previous research which has shown that nature‐based activities improved physical health (e.g., [87]), but does suggest that natural environments are often a facilitator for physical activity and walking in particular [88]. The discrepancy might be because the pace of walking might have been compromised by the challenges of the terrain, making walking slower or more difficult within the natural environment. This could have lengthened the overall walking time while not contributing to relatively fast‐paced walking considered important for improving health. Lower activity intensity due to intermittent movement could also contribute to the discrepancy. Exploring elements of nature such as wildlife, varied landscapes, and species [89] are attractive features that older people enjoy, and they are key features of parks. The tendency to pause and then resume walking might result in relatively lower activity intensity that did not translate to better physical health.

A further possibility is that the relevant outcomes were not measured. Nature engagement showed no association with any health or physical function measure here, but affective response may be where its effects are more readily detected. J. I. Kim and G. Kim [90] found that older adults reported gladness, happiness, reduced stress and a relaxed mind following exposure to a natural environment, with reported change roughly twofold higher in those over 70 than in those aged 60 to 69. Their exposure was curated, and their outcomes subjective, so the comparison is indicative rather than direct, but it suggests that measures capturing mood and affect might reveal associations that functional and general health indicators do not.

Taken together with the finding that natural environments were the most frequently reported location across trip purposes, this suggests that nature is largely experienced through activity rather than sought for its own sake. The design implication is specific: green space is better conceived as a multifunctional setting that hosts routine and social activity than as scenery or as a nature reserve. Provision that follows from this would prioritise seating at intervals, paths that connect to everyday destinations such as shops and community venues, surfaces that accommodate varied mobility, and space in which groups can gather, in preference to treating naturalness or biodiversity as the design objective in itself. Given that trips to engage with nature occurred closer to home than any other purpose, this provision matters most at the neighbourhood scale.

Utilitarian trip frequency was not associated with health scores, which might suggest that errands are inconsequential for wellbeing. Utilitarian trips were the most frequent in terms of trip purpose but had the shortest walking time, and significantly less than trips whose purpose was for nature visits or recreation/exercise. However, because of their relatively high frequency, even small changes to quotidian activities and their associated environments might accumulate benefit, leading to better physical and mental health outcomes. An example of this in a Japan‐based study used shopping as a way to help older people with mobility issues to rehabilitate physically and to gain better mobility [91]. These benefits were only significant when the programme was delivered as a group intervention instead of an individual intervention, highlighting the virtue of incorporating social encounters and interactions into utilitarian tasks for health and wellbeing benefits.

Besides focusing on the purpose of the trip itself as a strategy for enhancing health and wellbeing, improving the physical environment could be another approach. The highly travelled spaces within the neighbourhood, used particularly frequently for utilitarian trips, are inextricably linked to the physical, social, and affective neighbourhood environmental context, all of which contribute to a sense of attachment and wellbeing in older adults [92]. Although older adults’ usual range of travel may be limited, through frequent OOH trips they can develop an intimate knowledge of their local environment, which can increase their sense of control over “their” place [93]. Place attachment is evident in helping to meet higher psychological and spiritual needs [94] that contribute to wellbeing, although measures of this were not considered in the current study (and may not have offered significant results). In other studies, improvements in street design—such as more continuous pavements with nonslip surfaces and lighting and more frequent and visible seating and shelter—have been shown to increase older people’s confidence in performing daily activities independently [95], which could indirectly and positively impact the higher‐level psychological needs mentioned above.

Promoting and supporting social engagement as the main purpose of OOH trips could be an alternative strategy to promote positive health outcomes, despite being associated in our study with comparatively lower levels of physical activity. Engagement with people involved the shortest walking times among all trip purposes, yet a higher frequency of social trips was associated with significantly better perceived health. Our study showed relatively short walking times involved in trips to cafés and other people’s homes, suggesting that relatively sedentary social participation can still be salutary. This finding is consistent with results from a larger sample of older people in the same context, where social engagement was associated with enjoyment of outdoor activities [79], which might contribute to the pursuits being sustained and ultimately enhancing health and wellbeing. Other bodies of evidence also suggest that social interactions and support are important to incorporate in interventions to promote physical activity, reduce anxiety and depression, and treat other combinations of illness experienced by older people [96, 97]. This suggests that social connectivity, independent of levels of physical activity, is a critical pathway to wellbeing in later life and has direct implications for designing targeted interventions for the health and wellbeing of older people.

Frequency of car use was associated with better functional ability and, marginally, with higher quality of life, suggesting that motorised travel may support rather than substitute for engagement in later life within this sample. Access to a car has been associated with better self‐reported health and mental health among older people, particularly those living alone [98], and may extend the range of destinations available to people whose walking is constrained. It may equally indicate a reliance that leaves activity vulnerable to driving cessation, which has been linked to depression, anxiety and poorer general mental health, with outcomes shaped by the person’s resources, transport availability and social networks [99]. Time spent on public transport showed no association with any outcome in this study, although the small number of such trips means little should be inferred from this. Elsewhere, public transport use among older people in the UK has been linked to more frequent walking and active travel [100, 101] and to slower decline in walking speed [102], so the absence of an association here is more plausibly a feature of these data than an indication that the relationship is absent. Taken together, these observations suggest that no single mode carries the relationship between everyday mobility and wellbeing.

The study showed a positive association between average sitting time and perceived health but no significant results between the average walking time and perceived health. There are several potential explanations for this counterintuitive finding. The sample was highly active compared with national averages, with a high weekly walking time, which is likely to reduce variance associated with this activity and makes dose–response gradients harder to detect. This is supported by the significant positive correlation, as shown in Table 6, between average walking time and the average time spent outdoors. In this cohort, time spent sitting likely captured time spent in social or restorative contexts—conversing in cafés, resting in parks—where the affective and cognitive benefits of environment may outweigh any negative connotations of sedentariness. The distinction between spatial stillness, meaning not travelling between places, and bodily stillness, meaning not moving the body, offers a useful lens here [103]. A participant sitting in a café is spatially still but has travelled to be there, and the setting itself may prompt bodily movement that a comparable period at home would not, since levels of bodily movement have been found to be lower inside the home than in places outside it. Sitting recorded within an OOH trip therefore indexes something different from sedentary time in general, which may explain why the measure behaved unexpectedly in relation to perceived health.

### 4.1. Limitations

This study has limitations, the most evident of which is that the sample is relatively active, with high values for weekly walking time, which may limit generalisability and create ceiling effects that obscure associations between walking and wellbeing. However, similar self‐selection bias toward more active older adults has been noted in comparable diary studies [65], likely reflecting the characteristics of individuals who are willing to participate in detailed, longitudinal data collection. Crucially, despite the high average activity levels, we observed reasonable variation in IADL scores within our sample. This variation is valuable, as it demonstrates how individuals can maintain high levels of activity and independence with low IADL scores but nonetheless can be relatively active even while experiencing functional limitations (higher IADL scores), partially mitigating the bias in our sample. Older adults were defined here as those aged 50 and over to reflect differences in healthy life expectancy across Scotland, particularly in more deprived areas. In practice the sample is skewed considerably older, with two participants aged 50 to 59 and 16 aged 70 or over, so comparisons with studies using a 65 or 70 threshold are less affected by the difference in criteria than the definition alone would suggest.

Participants who completed fewer diaries reported poorer functional ability, lower quality of life and lower perceived health than those completing three. Since the trip‐level analyses draw more heavily on participants contributing more diaries, the recorded trips over‐represent those in better health, which compounds the general activity bias of the sample noted above. The sensitivity analysis, in which each participant contributed a single trip, addresses the differential weighting but not the composition of the sample itself. The findings are therefore better read as describing the OOH activity of relatively healthy older adults than as representative of the age group as a whole.

To align trip data with person‐level health measures, we aggregated to the participant level, a necessary step that reduces within‐person variability and may conceal nonlinear effects of purpose, place, or distance. Activity characteristics and durations were self‐reported and thus susceptible to recall or classification error, and the broad category of “sitting” within a trip does not differentiate between passive sedentariness and socially or cognitively engaging seated activities.

Activity locations were coded using an established deductive scheme shared with the wider study, which differentiates natural settings more finely than built ones. Coding a degree of naturalness across all locations would allow the character of settings to be treated on a single continuum, though it would require a level of locational detail beyond that recorded here.

The trip‐level analyses centre on walking time. Public transport was recorded on 58 of 742 trips, too few to support comparison across purpose and location categories, and person‐level metrics were used instead. Car use was recorded on 292 trips and could have supported trip‐level analysis but was not examined in this way because the study set out to explore whether more physically active OOH activity is associated with better health and wellbeing and because extending the trip‐level tests to further modes would have substantially increased the number of comparisons made within a small sample. A fuller account of how motorised travel is patterned by purpose and setting remains to be given.

Finally, while data were collected across more than one time point, the correlational analyses linking activity aggregates to health were cross‐sectional at the person level and cannot establish causal direction. Several unmeasured factors may also have shaped the patterns observed. Diaries were completed in both winter and summer, and seasonal conditions are known to affect how far and how often older people go out. Step counts among older adults peak at moderate temperatures and decline in both colder and warmer conditions, and fall with increasing precipitation, while ice and darkness are among the most significant deterrents to winter walking, with fear of falling reducing outdoor activity independently of physical capacity. A participant completing a diary in February would therefore be expected to record fewer and shorter trips than the same participant in July, and because participants contributed diaries in different combinations of seasons, this variation is embedded in the data without being modelled. At a finer scale, microclimatic conditions at particular locations, including exposure to wind, availability of shade and shelter and surface conditions underfoot, plausibly influence which settings are used and for how long and would bear most directly on the comparisons of walking time across locations. The distribution of amenities around each participant’s home determines whether utilitarian destinations are reachable on foot at all, which may account for some of the variation in walking time on trips of that purpose.

## 5. Conclusion

This study utilised a diary‐based methodology to investigate the socio‐ecological characteristics of OOH activities that older people living in their own homes engaged in daily, the associated environmental settings and their association with physical and overall health and wellbeing.

Results confirmed previous research showing that higher levels of OOH trips for recreation correlated with a better quality of life and physical function, and higher levels of OOH trips for social engagement correlated positively with self‐reported health.

Utilitarian trips were the most frequently reported trip purposes, with relatively shorter walking times and a more proximate distance to participants’ homes than for other types of trips. This suggests that older people are likely to develop place attachment and confidence when performing these quotidian activities and engaging with the environmental context in which they take place. If the environment can be designed to facilitate such higher‐order psychological needs, not captured in this study, they could contribute to better wellbeing indirectly.

Future work should employ more objective methods to assess the intensity and pace of walking, particularly in nature, to clarify its true impact on physical health. Samples of less active older adults should also be sought to represent the diversity of the wider population. Longitudinal and intervention studies are necessary to establish the causality of these associations and to measure the cumulative impact of small‐scale, neighbourhood‐focused environmental changes.

## Funding

This research was funded by the Legal and General Group (research grant to establish the independent Advanced Care Research Centre at University of Edinburgh). The views expressed are those of the authors and not necessarily those of Legal and General.

## Disclosure

The funder had no role in conduct of the study, interpretation or the decision to submit for publication. This work forms part of a wider study of older adults’ personal projects and their local outdoor environments, hosted by the Advanced Care Research Centre. Findings from the qualitative strand of that study are reported separately [104].

## Conflicts of Interest

The authors declare no conflicts of interest.

## Supporting Information

Additional supporting information can be found online in the Supporting Information section.

## Supporting information


**Supporting Information** Appendix A Activity diary booklet—The diary booklet provides structured space for participants to record their activities over a 7‐day period. Participants are encouraged to write about their feelings and spontaneously events, such as stopping for coffee or meeting a friend.

## Data Availability

The data that support the findings of this study are available on request from the corresponding author. The data are not publicly available due to privacy or ethical restrictions.

## References

[1] World Health Organization , World Report on Ageing and Health, 2015, https://iris.who.int/handle/10665/186463.

[2] Milligan C. , There’s no Place Like Home: Place and Care in an Ageing Society, 2009, Routledge.

[3] van Dijk H. M. , Cramm J. M. , van Exel J. , and Nieboer A. P. , The Ideal Neighbourhood for Ageing in Place as Perceived by Frail and Non-Frail Community-Dwelling Older People, Ageing and Society. (2015) 35, no. 8, 1771–1795, 10.1017/s0144686x14000622.

[4] Wiles J. L. , Leibing A. , Guberman N. , Reeve J. , and Allen R. E. , The Meaning of “Aging in Place” to Older People, The Gerontologist. (2012) 52, no. 3, 357–366, 10.1093/geront/gnr098. 21983126

[5] Hwang E. , Cummings L. , Sixsmith A. , and Sixsmith J. , Impacts of Home Modifications on Aging-in-Place, Journal of Housing for the Elderly. (2011) 25, no. 2, 246–257, 10.1080/02763893.2011.595611.

[6] Tümer A. , Dönmez S. , Gümüşsoy S. , and Balkaya N. A. , The Relationship Among Aging in Place, Loneliness, and Life Satisfaction in the Elderly in Turkey, Perspectives in Psychiatric Care. (2022) 58, no. 2, 822–829, 10.1111/ppc.12855. 34018200

[7] Wang Z. , Shepley M. M. , and Rodiek S. D. , Aging in Place at Home Through Environmental Support of Physical Activity: An Interdisciplinary Conceptual Framework and Analysis, Journal of Housing for the Elderly. (2012) 26, no. 4, 338–354, 10.1080/02763893.2011.625289.

[8] Martin P. , Kelly N. , Kahana B. et al., Defining Successful Aging: A Tangible or Elusive Concept?, The Gerontologist. (2015) 55, no. 1, 14–25, 10.1093/geront/gnu044. PMC4542894 24840916

[9] Michel J.-P. and Sadana R. , Healthy Aging Concepts and Measures, Journal of the American Medical Directors Association. (2017) 18, no. 6, 460–464, 10.1016/j.jamda.2017.03.008. 28479271

[10] Havighurst R. J. , Williams R. H. , Tibbitts C. , and Donahue W. , Successful Aging, Processes of Aging: Social and Psychological Perspectives, 1963, 1, Atherton Press, 299–320.

[11] Rowe J. W. and Kahn R. L. , Successful Aging, The Gerontologist. (1997) 37, no. 4, 433–440. 10.1093/geront/37.4.433 9279031

[12] World Health Organization , Active Ageing: A Policy Framework (WHO/NMH/NPH/02.8), 2002, https://iris.who.int/handle/10665/67215. 12040973

[13] World Health Organization , UN Decade of Healthy Ageing: Plan of Action, 2020, https://www.who.int/initiatives/decade-of-healthy-ageing.

[14] Sprod J. , Ferrar K. , Olds T. , and Maher C. , Changes in Sedentary Behaviours Across the Retirement Transition: A Systematic Review, Age and Ageing. (2015) 44, no. 6, 918–925, 10.1093/ageing/afv140. 26504115

[15] Sprod J. , Olds T. , Brown W. et al., Changes in Use of Time Across Retirement: A Longitudinal Study, Maturitas. (2017) 100, 70–76, 10.1016/j.maturitas.2017.02.018. 28539179

[16] Strazdins L. , Welsh J. , Korda R. , Broom D. , and Paolucci F. , Not all Hours are Equal: Could Time be a Social Determinant of Health?, Sociology of Health & Illness. (2016) 38, no. 1, 21–42, 10.1111/1467-9566.12300. 26174027

[17] Weir D. R. , Waite L. J. , Wong R. , and Freedman V. A. , New Measures and New Designs in Demography of Aging Research: Proceedings of a Workshop, 2018, National Academies Press.

[18] Chow H. W. , Chen H. C. , and Lin L. L. , Association Between Out‐of‐Home Trips and Older Adults’ Functional Fitness, Geriatrics and Gerontology International. (2014) 14, no. 3, 596–604, 10.1111/ggi.12143. 24007358

[19] Harada K. , Lee S. , Park H. et al., Going Outdoors and Cognitive Function Among Community-Dwelling Older Adults: Moderating Role of Physical Function, Geriatrics and Gerontology International. (2016) 16, no. 1, 65–73, 10.1111/ggi.12437. 25597479

[20] Harada K. , Lee S. , Lee S. et al., Objectively‐Measured Outdoor Time and Physical and Psychological Function Among Older Adults, Geriatrics and Gerontology International. (2017) 17, no. 10, 1455–1462, 10.1111/ggi.12895. 27633728

[21] Inoue K. , Shono T. , and Matsumoto M. , Absence of Outdoor Activity and Mortality Risk in Older Adults Living at Home, Journal of Aging and Physical Activity. (2006) 14, no. 2, 203–211, 10.1123/japa.14.2.203. 19462550

[22] Brajša-Žganec A. , Merkaš M. , and Šverko I. , Quality of Life and Leisure Activities: How do Leisure Activities Contribute to Subjective Well-Being?, Social Indicators Research. (2011) 102, no. 1, 81–91, 10.1007/s11205-010-9724-2.

[23] Fernández-Ballesteros R. , Environmental Conditions, Health and Satisfaction Among the Elderly: Some Empirical Results, Psicothema. (2001) 13, no. 1, 41–49.

[24] Harlow R. E. and Cantor N. , Still Participating After All These Years: A Study of Life Task Participation in Later Life, Journal of Personality and Social Psychology. (1996) 71, no. 6, 1235–1248, 10.1037/0022-3514.71.6.1235. 8979389

[25] Hao Y. , Productive Activities and Psychological Well-Being Among Older Adults, Journals of Gerontology Series B: Psychological Sciences and Social Sciences. (2008) 63, no. 2, S64–S72, 10.1093/geronb/63.2.s64. 18441271

[26] McMunn A. , Nazroo J. , Wahrendorf M. , Breeze E. , and Zaninotto P. , Participation in Socially-Productive Activities, Reciprocity and Wellbeing in Later Life: Baseline Results in England, Ageing and Society. (2009) 29, no. 5, 765–782, 10.1017/s0144686x08008350.

[27] Matz-Costa C. , Besen E. , James J. B. , and Pitt-Catsouphes M. , Differential Impact of Multiple Levels of Productive Activity Engagement on Psychological Well-Being in Middle and Later Life, The Gerontologist. (2014) 54, no. 2, 277–289, 10.1093/geront/gns148. 23213083

[28] Klumb P. L. , Benefits From Productive and Consumptive Activities: Results From the Berlin Aging Study, Social Indicators Research. (2004) 67, no. 1–2, 107–127, 10.1023/b:soci.0000007336.64239.a6.

[29] Hunter K. I. and Linn M. W. , Psychosocial Differences Between Elderly Volunteers and Non-Volunteers, The International Journal of Aging and Human Development. (1981) 12, no. 3, 205–213, 10.2190/0h6v-qppp-7jk4-lr38. 7216525

[30] Patterson I. , Participation in Leisure Activities by Older Adults After a Stressful Life Event: The Loss of a Spouse, The International Journal of Aging and Human Development. (1996) 42, no. 2, 123–142, 10.2190/tg1m-75cb-pl27-r6g3. 8859495

[31] Janke M. C. , Payne L. L. , and Van Puymbroeck M. , The Role of Informal and Formal Leisure Activities in the Disablement Process, The International Journal of Aging and Human Development. (2008) 67, no. 3, 231–257, 10.2190/ag.67.3.c. 19049245

[32] Janke M. , Davey A. , and Kleiber D. , Modeling Change in Older Adults’ Leisure Activities, Leisure Sciences. (2006) 28, no. 3, 285–303, 10.1080/01490400600598145.

[33] Jopp D. S. and Hertzog C. , Assessing Adult Leisure Activities: An Extension of a Self-Report Activity Questionnaire, Psychological Assessment. (2010) 22, no. 1, 108–120, 10.1037/a0017662. PMC2841313 20230157

[34] Lee S. , Koffer R. E. , Sprague B. N. , Charles S. T. , Ram N. , and Almeida D. M. , Activity Diversity and Its Associations With Psychological Well-Being Across Adulthood, The Journals of Gerontology: Serie Bibliographique. (2018) 73, no. 6, 985–995. 10.1093/geronb/gbw118 PMC6454790 27621306

[35] Barnett D. W. , Barnett A. , Nathan A. , Van Cauwenberg J. , and Cerin E. , Built Environmental Correlates of Older Adults’ Total Physical Activity and Walking: A Systematic Review and Meta-Analysis, International Journal of Behavioral Nutrition and Physical Activity. (2017) 14, no. 1, 10.1186/s12966-017-0558-z. PMC5547528 28784183

[36] Cerin E. , Nathan A. , Van Cauwenberg J. , Barnett D. W. , and Barnett A. , The Neighbourhood Physical Environment and Active Travel in Older Adults: A Systematic Review and Meta-Analysis, International Journal of Behavioral Nutrition and Physical Activity. (2017) 14. 10.1186/s12966-017-0471-5 PMC5294838 28166790

[37] Rachele J. N. , Sugiyama T. , Davies S. et al., Neighbourhood Built Environment and Physical Function Among Mid-to-Older Aged Adults: A Systematic Review, Health & Place. (2019) 58, 10.1016/j.healthplace.2019.05.015. 31176106

[38] Hirsch J. A. , Winters M. , Ashe M. C. , Clarke P. J. , and McKay H. A. , Destinations That Older Adults Experience Within Their GPS Activity Spaces: Relation to Objectively Measured Physical Activity, Environment and Behavior. (2014) 48, no. 1, 55–77, 10.1177/0013916515607312. PMC4714356 26783370

[39] Perchoux C. , Chaix B. , Cummins S. , and Kestens Y. , Conceptualization and Measurement of Environmental Exposure in Epidemiology: Accounting for Activity Space Related to Daily Mobility, Health & Place. (2013) 21, 86–93, 10.1016/j.healthplace.2013.01.005. 23454664

[40] Rotenberg S. , Fritz H. , Chippendale T. , Lysack C. , and Cutchin M. , Out-of-Home Activities and Health in Older Adults: A Scoping Review, OTJR: Occupation, Participation and Health. (2021) 41, no. 4, 285–298, 10.1177/15394492211023192. PMC8479153 34148393

[41] Shoval N. , Auslander G. , Cohen-Shalom K. , Isaacson M. , Landau R. , and Heinik J. , What Can We Learn About the Mobility of the Elderly in the GPS Era?, Journal of Transport Geography. (2010) 18, no. 5, 603–612, 10.1016/j.jtrangeo.2010.03.012.

[42] Winters M. , Voss C. , Ashe M. C. , Gutteridge K. , McKay H. , and Sims-Gould J. , Where Do They Go and How Do They Get There? Older Adults’ Travel Behaviour in a Highly Walkable Environment, Social Science & Medicine. (2015) 133, 304–312, 10.1016/j.socscimed.2014.07.006. 25017579

[43] Schwanen T. and Ziegler F. , Wellbeing, Independence and Mobility: An Introduction, Ageing and Society. (2011) 31, no. 5, 719–733, 10.1017/s0144686x10001467.

[44] Schwanen T. , Banister D. , and Bowling A. , Independence and Mobility in Later Life, Geoforum. (2012) 43, no. 6, 1313–1322, 10.1016/j.geoforum.2012.04.001.

[45] Metz D. H. , Mobility of Older People and Their Quality of Life, Transport Policy. (2000) 7, no. 2, 149–152, 10.1016/s0967-070x(00)00004-4.

[46] Elliott J. and Green J. , Are Physical Activity and Everyday Mobility Independently Associated With Quality of Life at Older Age?, Aging and Health Research. (2024) 4, no. 3, 10.1016/j.ahr.2024.100204. PMC11413527 39309682

[47] Brookfield K. , Ward Thompson C. , and Scott I. , The Uncommon Impact of Common Environmental Details on Walking in Older Adults, International Journal of Environmental Research and Public Health. (2017) 14, no. 2, 10.3390/ijerph14020190. PMC5334744 28216597

[48] Olsen J. R. , Whitley E. , Long E. et al., Individual, Social and Area Level Factors Associated With Older People’s Walking: Analysis of an UK Household Panel Study (Understanding Society), Social Science & Medicine. (2024) 358, 10.1016/j.socscimed.2024.117083. 39226800

[49] Bowling A. , Measuring the Quality of Later Life, The New Dynamics of Ageing, 2018, Policy Press, 81–102.

[50] Peel C. , Sawyer Baker P. , Roth D. L. , Brown C. J. , Brodner E. V. , and Allman R. M. , Assessing Mobility in Older Adults: The UAB Study of Aging Life-Space Assessment, Physical Therapy. (2005) 85, no. 10, 1008–1119, 10.1093/ptj/85.10.1008. 16180950

[51] Tsai L. T. , Portegijs E. , Rantakokko M. et al., The Association Between Objectively Measured Physical Activity and Life-Space Mobility Among Older People, Scandinavian Journal of Medicine & Science in Sports. (2015) 25, no. 4, e368–e373, 10.1111/sms.12337. 26152855

[52] Viljanen A. , Mikkola T. M. , Rantakokko M. , Portegijs E. , and Rantanen T. , The Association Between Transportation and Life-Space Mobility in Community-Dwelling Older People With or Without Walking Difficulties, Journal of Aging and Health. (2016) 28, no. 6, 1038–1054, 10.1177/0898264315618919. 26613926

[53] Chung J. , Ozkaynak M. , and Demiris G. , Examining Daily Activity Routines of Older Adults Using Workflow, Journal of Biomedical Informatics. (2017) 71, 82–90, 10.1016/j.jbi.2017.05.010. 28529189

[54] McInnes L. , Jones E. , Rochester L. et al., Mobility in Community Dwelling Older Adults: Predicting Successful Mobility Using an Instrumented Battery of Novel Measures, The Journal of Frailty & Aging. (2020) 9, no. 2, 68–73, 10.14283/jfa.2019.35. PMC12275692 32259179

[55] Tsai L. T. , Rantakokko M. , Rantanen T. , Viljanen A. , Kauppinen M. , and Portegijs E. , Objectively Measured Physical Activity and Changes in Life-Space Mobility Among Older People, Journals of Gerontology. Series A, Biological Sciences and Medical Sciences. (2016) 71, no. 11, 1466–1471, 10.1093/gerona/glw042. 26975981

[56] Anderson M. and Perrin A. , Technology Use Among Seniors, 2017, Pew Research Center for Internet & Technology.

[57] Pew Research Center , Demographics of Mobile Device Ownership and Adoption in the United States, 2025, https://www.pewresearch.org/internet/fact-sheet/mobile/.

[58] Ofcom , Online Nation: 2025 Report, 2025, https://www.ofcom.org.uk/siteassets/resources/documents/research-and-data/online-research/online-nation/2025/online-nations-report-2025.pdf.

[59] Krishnasamy C. , Unsworth C. , and Howie L. , The Patterns of Activity, and Transport to Activities Among Older Adults in Singapore, Hong Kong Journal of Occupational Therapy. (2011) 21, no. 2, 80–87, 10.1016/j.hkjot.2011.12.002.

[60] Sabbath E. L. , Matz-Costa C. , Rowe J. W. et al., Social Predictors of Active Life Engagement: A Time-Use Study of Young-Old French Adults, Research on Aging. (2016) 38, no. 8, 864–893, 10.1177/0164027515609408. 26449627

[61] Little B. R. , Personal Projects: A Rationale and Method for Investigation, Environment and Behavior. (1983) 15, no. 3, 273–309, 10.1177/0013916583153002.

[62] Little B. R. , Personal Project Pursuit: Goals, Action, and Human Flourishing, 2007, Lawrence Erlbaum Associates.

[63] Wallenius M. , Personal Projects in Everyday Places: Perceived Supportiveness of the Environment and Psychological Well-Being, Journal of Environmental Psychology. (1999) 19, no. 2, 131–143, 10.1006/jevp.1998.0118.

[64] Kelly G. A. , The Psychology of Personal Constructs, 1955, Norton.

[65] Roe J. , Blythe M. , Oliver C. , and Roe A. , Flourishing ‘Older-Old’ (80+) Adults: Personal Projects and Their Enabling Places, Wellbeing, Space and Society. (2022) 3, 10.1016/j.wss.2022.100072.

[66] McKenna K. , Broome K. , and Liddle J. , What Older People Do: Time Use and Exploring the Link Between Role Participation and Life Satisfaction in People Aged 65 Years and Over, Australian Occupational Therapy Journal. (2007) 54, no. 4, 273–284, 10.1111/j.1440-1630.2007.00642.x.

[67] Björklund C. , Erlandsson L. K. , Lilja M. , and Gard G. , Temporal Patterns of Daily Occupations Related to Older Adults’ Health in Northern Sweden, Journal of Occupational Science. (2014) 22, no. 2, 127–145, 10.1080/14427591.2014.913330.

[68] Björklund C. , Gard G. , Lilja M. , and Erlandsson L. K. , Temporal Patterns of Daily Occupations Among Older Adults in Northern Sweden, Journal of Occupational Science. (2013) 21, no. 2, 143–160, 10.1080/14427591.2013.790666.

[69] Hunt E. and McKay E. A. , A Scoping Review of Time-Use Research in Occupational Therapy and Occupational Science, Scandinavian Journal of Occupational Therapy. (2015) 22, no. 1, 1–12, 10.3109/11038128.2014.934918. 25135710

[70] Jacelon C. S. and Imperio K. , Participant Diaries as a Source of Data in Research With Older Adults, Qualitative Health Research. (2005) 15, no. 7, 991–997, 10.1177/1049732305278603. 16093375

[71] Milligan C. , Bingley A. , and Gatrell A. , Digging Deep: Using Diary Techniques to Explore the Place of Health and Well-Being Amongst Older People, Social Science & Medicine. (2005) 61, no. 9, 1882–1892, 10.1016/j.socscimed.2005.04.002. 15913863

[72] Mao Y. and Kubo T. , Everyday Activities of Older Adults in Tianjin, China: Coupling Constraints, Gendered Mobilities and Social Context, Behavioral Sciences. (2023) 13, no. 12, 10.3390/bs13120996. PMC10740803 38131852

[73] Mochcovitch D. , Jones A. , Goutte J. et al., Exploring Mobility Patterns and Social Health of Older Canadians Living at Home to Inform Decision Aids About Housing: A Mixed-Methods Study, PLoS One. (2025) 20, no. 4, 10.1371/journal.pone.0320876. PMC12005502 40245024

[74] Sheppard C. L. , Myers A. M. , and Hesjedahl S. , Using Travel Diaries to Examine the Extent to Which Older Adult Centres are a Focal Point for Recreation and Social Activities, Canadian Journal on Aging. (2023) 42, no. 3, 495–505, 10.1017/s0714980823000120. 37439086

[75] Zeitler E. , Buys L. , Aird R. , and Miller E. , Mobility and Active Ageing in Suburban Environments: Findings From In-Depth Interviews and Person-Based GPS Tracking, Current Gerontology and Geriatrics Research. (2012) 2012, 10.1155/2012/257186. PMC3533602 23346108

[76] Foong H. F. , Lim S. Y. , Koris R. , and Haron S. A. , Time-Use and Mental Health in Older Adults: A Scoping Review, International Journal of Environmental Research and Public Health. (2021) 18, no. 9, 10.3390/ijerph18094459. PMC8122809 33922295

[77] Ellegård K. , A Time-Geographical Approach to the Study of Everyday Life of Individuals–A Challenge of Complexity, GeoJournal. (1999) 48, no. 3, 167–175, 10.1023/a:1007071407502.

[78] Stanley M. , Richard A. , and Williams S. , Older Peoples’ Perspectives on Time Spent Alone, Australian Occupational Therapy Journal. (2017) 64, no. 3, 235–242, 10.1111/1440-1630.12353. 27966222

[79] Tong K. , Pearce C. , Kingston A. , Berry K. , and Ward Thompson C. , Older Adults’ Outdoor Activities and Quality of Life: A Personal Projects Approach, Journal of Aging and Environment. (2025) 1–27, 10.1080/26892618.2025.2552794.

[80] Pearce J. , Witten K. , Hiscock R. , and Blakely T. , Are Socially Disadvantaged Neighbourhoods Deprived of Health-Related Community Resources?, International Journal of Epidemiology. (2007) 36, no. 2, 348–355, 10.1093/ije/dyl267. 17182634

[81] Ward Thompson C. , Curl A. , Aspinall P. , Alves S. , and Zuin A. , Do Changes to the Local Street Environment Alter Behaviour and Quality of Life of Older Adults? The ‘DIY Streets’ Intervention, British Journal of Sports Medicine. (2014) 48, no. 13, 1059–1065, 10.1136/bjsports-2012-091718. PMC4078749 23242959

[82] Jette A. M. , Functional Disability and Rehabilitation of the Aged, Topics in Geriatric Rehabilitation. (1986) 1, no. 3, 1–8, 10.1097/00013614-198604000-00003.

[83] Curl A. , Thompson C. W. , Alves S. , and Aspinall P. , Outdoor Environmental Supportiveness and Older People’s Quality of Life: A Personal Projects Approach, Journal of Housing for the Elderly. (2016) 30, no. 1, 1–17, 10.1080/02763893.2015.1087925.

[84] Strain T. , Kelly P. , Gibb R. , Allison M. , Mutrie N. , and Murphy M. , Is Scotland Walking in the Right Direction? A Cross-Sectional Analysis of Trends in Walking by Socioeconomic Status, Journal of Physical Activity and Health. (2024) 21, no. 7, 707–716, 10.1123/jpah.2023-0635. 38688465

[85] Kim J. I. , Sociology of Longevity: Socioecological Factors of Survival Probability, 2022, Cambridge Scholars Publishing.

[86] Currie M. , Colley K. , and Irvine K. N. , Outdoor Recreation for Older Adults in Scotland: Qualitatively Exploring the Multiplicity of Constraints to Participation, International Journal of Environmental Research and Public Health. (2021) 18, no. 14, 10.3390/ijerph18147705. PMC8307115 34300154

[87] Struthers N. A. , Guluzade N. A. , Zecevic A. A. , Walton D. M. , and Gunz A. , Nature-Based Interventions for Physical Health Conditions: A Systematic Review and Meta-Analysis, Environmental Research. (2024) 258, 10.1016/j.envres.2024.119421. 38876421

[88] Keskinen K. E. , Rantakokko M. , Suomi K. , Rantanen T. , and Portegijs E. , Nature as a Facilitator for Physical Activity: Defining Relationships Between the Objective and Perceived Environment and Physical Activity Among Community-Dwelling Older People, Health & Place. (2018) 49, 111–119, 10.1016/j.healthplace.2017.12.003. 29306140

[89] Kou R. , Hunter R. F. , Cleland C. , and Ellis G. , Physical Environmental Factors Influencing Older Adults’ Park Use: A Qualitative Study, Urban Forestry and Urban Greening. (2021) 65, 10.1016/j.ufug.2021.127353.

[90] Kim J. I. and Kim G. , Exploring Subjective Mental Health Change in Older Adults After Experiencing an Immersive Virtual Reality (IVR)-Based Natural Environment, Journal of Applied Gerontology. (2025) 44, no. 3, 364–376, 10.1177/07334648241272042. 39150382

[91] Mouri N. , Ohta R. , and Sano C. , Effects of Shopping Rehabilitation on Older People’s Daily Activities, International Journal of Environmental Research and Public Health. (2022) 19, no. 1, 10.3390/ijerph19010569. PMC8744656 35010839

[92] Wiles J. L. , Allen R. E. S. , Palmer A. J. , Hayman K. J. , Keeling S. , and Kerse N. , Older People and Their Social Spaces: A Study of Well-Being and Attachment to Place in Aotearoa New Zealand, Social Science & Medicine. (2009) 68, no. 4, 664–671, 10.1016/j.socscimed.2008.11.030. 19110361

[93] Holland C. , Kellaher L. , Peace S. M. et al., Walker A. , Getting Out and About, Understanding Quality of Life in Old Age, 2005, Open University Press, 49–63.

[94] Sun Y. , Ng M. K. , Chao T. Y. S. , He S. , and Mok S. H. , The Impact of Place Attachment on Well-Being for Older People in High-Density Urban Environment: A Qualitative Study, Journal of Aging & Social Policy. (2024) 36, no. 2, 241–261, 10.1080/08959420.2022.2111168. 35957602

[95] van Hoven B. and Meijering L. , Mundane Mobilities in Later Life-Exploring Experiences of Everyday Trip-Making by Older Adults in a Dutch Urban Neighbourhood, Research in Transportation Business & Management. (2019) 30, 10.1016/j.rtbm.2019.100375.

[96] Tcymbal A. , Abu-Omar K. , Hartung V. et al., Interventions Simultaneously Promoting Social Participation and Physical Activity in Community Living Older Adults: A Systematic Review, Frontiers in Public Health. (2022) 10, 10.3389/fpubh.2022.1048496. PMC9768837 36568739

[97] Menhas R. , Yang L. , Saqib Z. A. , Younas M. , and Saeed M. M. , Does Nature-Based Social Prescription Improve Mental Health Outcomes? A Systematic Review and Meta-Analysis, Frontiers in Public Health. (2024) 12, 10.3389/fpubh.2024.1228271. PMC10999630 38590811

[98] Doebler S. , Access to a Car and the Self-Reported Health and Mental Health of People Aged 65 and Older in Northern Ireland, Research on Aging. (2016) 38, no. 4, 453–476, 10.1177/0164027515590424. 26055983

[99] Liddle J. , Scott T. L. , Hoyle M. , Salmon A. , Gustafsson L. , and Pachana N. A. , The Mental Health Outcomes of Driving Cessation for Older People: A Systematic Review, International Psychogeriatrics. (2024) 10.1016/j.inpsyc.2024.100020.

[100] Coronini-Cronberg S. , Millett C. , Laverty A. A. , and Webb E. , The Impact of a Free Older Persons’ Bus Pass on Active Travel and Regular Walking in England, American Journal of Public Health. (2012) 102, no. 11, 2141–2148, 10.2105/ajph.2012.300946. PMC3477933 22994251

[101] Webb E. , Netuveli G. , and Millett C. , Free Bus Passes, Use of Public Transport and Obesity Among Older People in England, Journal of Epidemiology & Community Health. (2012) 66, no. 2, 176–180, 10.1136/jech.2011.133165. 21911850

[102] Rouxel P. , Webb E. , and Chandola T. , Does Public Transport Use Prevent Declines in Walking Speed Among Older Adults Living in England? A Prospective Cohort Study, BMJ Open. (2017) 7, no. 9, 10.1136/bmjopen-2017-017702. PMC5652510 28963309

[103] Sattari Z. , Meijering L. , and Weitkamp G. , A More Realistic Picture of Older Adults’ Activity Spaces: Combining Spatial and Bodily Micro-Movements, Health & Place. (2026) 98, 10.1016/j.healthplace.2026.103625. 41643595

[104] Pearce C. , Ward Thompson C. , and Berry K. , This is Where I Can be Myself: Places That Enable Flourishing in Older Age, Health & Place. (2026) 98, 10.1016/j.healthplace.2026.103624. 41678871

[105] Scottish Executive , Scottish Index of Multiple Deprivation 2004: Summary Technical Report, 2004, Scottish Executive.

