# Supplementary material for: Everyday Out‐of‐Home Activities and Healthy Ageing: A Diary‐Based Analysis of Activity Locations, Purposes and Characteristics and Their Relevance to Environmental Design for Ageing‐in‐Place
Source: J Aging Res. 2026 Oct 19;2026:3428651. doi: 10.1155/jare/3428651 (PMC13680858; doi:10.1155/jare/3428651)
Supplement: Supplementary file 1 [file JARE-2026-3428651-s001.docx]

**Supplementary Materials**

**Appendix A Activity diary booklet**

# **Activity Diary**

Thank you for volunteering to take part in the Advanced Care Research Centre research project carried out by researchers at The University of Edinburgh.

By filling in this activity diary you will help us better understand the outdoor activities of people in older age.

**Start date: _________________**

**End date: __________________**

Name:

Your email:

Your telephone number:

Please remember to:

- Fill in your diary in at the end of each day. There is space to report up to four outings per day.

And please…

- Write whatever you feel could help us understand the activities you do outside your home each day
- Don’t worry if you are not going out every day

**Day 1**

Time of day: Morning / Lunch / Afternoon / Evening (please circle)

I went out of the house at: : (specify time)

I was (tick all that apply) : ❒ Alone	❒ With my partner	❒ With a relative	❒ With a friend	❒ Walking a dog	❒ Other (please specify):

I went to (specify the location):

This location is:

❒ Outside my home (e.g. porch, garage, garden)

❒ Within my local neighbourhood (less than ½ mile away)

❒ Outside my local neighbourhood but within town (less than 5 miles)

❒ Outside town (less or more than 10 miles)

The main purpose of going out was:

When you were outside about how long did you spend:

Walking………………………. In a car………………………………………….

Sitting…………………………..Using public transport…………………..

Cycling………………………….Other (please specify)………….…………

While I was out I also did:

*e.g. bumped into a friend, stopped for a coffee*

I went back into the house at: : (specify time)

**Day 1**

Time of day: Morning / Lunch / Afternoon / Evening (please circle)

I went out of the house at: : (specify time)

I was (tick all that apply) : ❒ Alone	❒ With my partner	❒ With a relative	❒ With a friend	❒ Walking a dog	❒ Other (please specify):

I went to (specify the location):

This location is:

❒ Outside my home (e.g. porch, garage, garden)

❒ Within my local neighbourhood (less than ½ mile away)

❒ Outside my local neighbourhood but within town (less than 5 miles)

❒ Outside town (less or more than 10 miles)

The main purpose of going out was:

When you were outside about how long did you spend:

Walking………………………. In a car………………………………………….

Sitting…………………………..Using public transport…………………..

Cycling………………………….Other……………………………………………

While I was out I also did:

*e.g. bumped into a friend, stopped for a coffee*

I went back into the house at: : (specify time)

**Day 1**

Time of day: Morning / Lunch / Afternoon / Evening (please circle)

I went out of the house at: : (specify time)

I was (tick all that apply) : ❒ Alone	❒ With my partner	❒ With a relative	❒ With a friend	❒ Walking a dog	❒ Other (please specify):

I went to (specify the location):

This location is:

❒ Outside my home (e.g. porch, garage, garden)

❒ Within my local neighbourhood (less than ½ mile away)

❒ Outside my local neighbourhood but within town (less than 5 miles)

❒ Outside town (less or more than 10 miles)

The main purpose of going out was:

When you were outside about how long did you spend:

Walking………………………. In a car………………………………………….

Sitting…………………………..Using public transport…………………..

Cycling………………………….Other……………………………………………

While I was out I also did:

*e.g. bumped into a friend, stopped for a coffee*

I went back into the house at: : (specify time)

**Day 1**

Time of day: Morning / Lunch / Afternoon / Evening (please circle)

I went out of the house at: : (specify time)

I was (tick all that apply) : ❒ Alone	❒ With my partner	❒ With a relative	❒ With a friend	❒ Walking a dog	❒ Other (please specify):

I went to (specify the location):

This location is:

❒ Outside my home (e.g. porch, garage, garden)

❒ Within my local neighbourhood (less than ½ mile away)

❒ Outside my local neighbourhood but within town (less than 5 miles)

❒ Outside town (less or more than 10 miles)

The main purpose of going out was:

When you were outside about how long did you spend:

Walking………………………. In a car………………………………………….

Sitting…………………………..Using public transport…………………..

Cycling………………………….Other……………………………………………

While I was out I also did:

*e.g. bumped into a friend, stopped for a coffee*

I went back into the house at: : (specify time)

**Day 2**

Time of day: Morning / Lunch / Afternoon / Evening (please circle)

I went out of the house at: : (specify time)

I was (tick all that apply) : ❒ Alone	❒ With my partner	❒ With a relative	❒ With a friend	❒ Walking a dog	❒ Other (please specify):

I went to (specify the location):

This location is:

❒ Outside my home (e.g. porch, garage, garden)

❒ Within my local neighbourhood (less than ½ mile away)

❒ Outside my local neighbourhood but within town (less than 5 miles)

❒ Outside town (less or more than 10 miles)

The main purpose of going out was:

When you were outside about how long did you spend:

Walking………………………. In a car………………………………………….

Sitting…………………………..Using public transport…………………..

Cycling………………………….Other……………………………………………

While I was out I also did:

*e.g. bumped into a friend, stopped for a coffee*

I went back into the house at: : (specify time)

**Day 2**

Time of day: Morning / Lunch / Afternoon / Evening (please circle)

I went out of the house at: : (specify time)

I was (tick all that apply) : ❒ Alone	❒ With my partner	❒ With a relative	❒ With a friend	❒ Walking a dog	❒ Other (please specify):

I went to (specify the location):

This location is:

❒ Outside my home (e.g. porch, garage, garden)

❒ Within my local neighbourhood (less than ½ mile away)

❒ Outside my local neighbourhood but within town (less than 5 miles)

❒ Outside town (less or more than 10 miles)

The main purpose of going out was:

When you were outside about how long did you spend:

Walking………………………. In a car………………………………………….

Sitting…………………………..Using public transport…………………..

Cycling………………………….Other……………………………………………

While I was out I also did:

*e.g. bumped into a friend, stopped for a coffee*

I went back into the house at: : (specify time)

**Day 2**

Time of day: Morning / Lunch / Afternoon / Evening (please circle)

I went out of the house at: : (specify time)

I was (tick all that apply) : ❒ Alone	❒ With my partner	❒ With a relative	❒ With a friend	❒ Walking a dog	❒ Other (please specify):

I went to (specify the location):

This location is:

❒ Outside my home (e.g. porch, garage, garden)

❒ Within my local neighbourhood (less than ½ mile away)

❒ Outside my local neighbourhood but within town (less than 5 miles)

❒ Outside town (less or more than 10 miles)

The main purpose of going out was:

When you were outside about how long did you spend:

Walking………………………. In a car………………………………………….

Sitting…………………………..Using public transport…………………..

Cycling………………………….Other……………………………………………

While I was out I also did:

*e.g. bumped into a friend, stopped for a coffee*

I went back into the house at: : (specify time)

**Day 2**

Time of day: Morning / Lunch / Afternoon / Evening (please circle)

I went out of the house at: : (specify time)

I was (tick all that apply) : ❒ Alone	❒ With my partner	❒ With a relative	❒ With a friend	❒ Walking a dog	❒ Other (please specify):

I went to (specify the location):

This location is:

❒ Outside my home (e.g. porch, garage, garden)

❒ Within my local neighbourhood (less than ½ mile away)

❒ Outside my local neighbourhood but within town (less than 5 miles)

❒ Outside town (less or more than 10 miles)

The main purpose of going out was:

When you were outside about how long did you spend:

Walking………………………. In a car………………………………………….

Sitting…………………………..Using public transport…………………..

Cycling………………………….Other……………………………………………

While I was out I also did:

*e.g. bumped into a friend, stopped for a coffee*

I went back into the house at: : (specify time)

**Day 3**

Time of day: Morning / Lunch / Afternoon / Evening (please circle)

I went out of the house at: : (specify time)

I was (tick all that apply) : ❒ Alone	❒ With my partner	❒ With a relative	❒ With a friend	❒ Walking a dog	❒ Other (please specify):

I went to (specify the location):

This location is:

❒ Outside my home (e.g. porch, garage, garden)

❒ Within my local neighbourhood (less than ½ mile away)

❒ Outside my local neighbourhood but within town (less than 5 miles)

❒ Outside town (less or more than 10 miles)

The main purpose of going out was:

When you were outside about how long did you spend:

Walking………………………. In a car………………………………………….

Sitting…………………………..Using public transport…………………..

Cycling………………………….Other……………………………………………

While I was out I also did:

*e.g. bumped into a friend, stopped for a coffee*

I went back into the house at: : (specify time)

**Day 3**

Time of day: Morning / Lunch / Afternoon / Evening (please circle)

I went out of the house at: : (specify time)

I was (tick all that apply) : ❒ Alone	❒ With my partner	❒ With a relative	❒ With a friend	❒ Walking a dog	❒ Other (please specify):

I went to (specify the location):

This location is:

❒ Outside my home (e.g. porch, garage, garden)

❒ Within my local neighbourhood (less than ½ mile away)

❒ Outside my local neighbourhood but within town (less than 5 miles)

❒ Outside town (less or more than 10 miles)

The main purpose of going out was:

When you were outside about how long did you spend:

Walking………………………. In a car………………………………………….

Sitting…………………………..Using public transport…………………..

Cycling………………………….Other……………………………………………

While I was out I also did:

*e.g. bumped into a friend, stopped for a coffee*

I went back into the house at: : (specify time)

**Day 3**

Time of day: Morning / Lunch / Afternoon / Evening (please circle)

I went out of the house at: : (specify time)

I was (tick all that apply) : ❒ Alone	❒ With my partner	❒ With a relative	❒ With a friend	❒ Walking a dog	❒ Other (please specify):

I went to (specify the location):

This location is:

❒ Outside my home (e.g. porch, garage, garden)

❒ Within my local neighbourhood (less than ½ mile away)

❒ Outside my local neighbourhood but within town (less than 5 miles)

❒ Outside town (less or more than 10 miles)

The main purpose of going out was:

When you were outside about how long did you spend:

Walking………………………. In a car………………………………………….

Sitting…………………………..Using public transport…………………..

Cycling………………………….Other……………………………………………

While I was out I also did:

*e.g. bumped into a friend, stopped for a coffee*

I went back into the house at: : (specify time)

**Day 3**

Time of day: Morning / Lunch / Afternoon / Evening (please circle)

I went out of the house at: : (specify time)

I was (tick all that apply) : ❒ Alone	❒ With my partner	❒ With a relative	❒ With a friend	❒ Walking a dog	❒ Other (please specify):

I went to (specify the location):

This location is:

❒ Outside my home (e.g. porch, garage, garden)

❒ Within my local neighbourhood (less than ½ mile away)

❒ Outside my local neighbourhood but within town (less than 5 miles)

❒ Outside town (less or more than 10 miles)

The main purpose of going out was:

When you were outside about how long did you spend:

Walking………………………. In a car………………………………………….

Sitting…………………………..Using public transport…………………..

Cycling………………………….Other……………………………………………

While I was out I also did:

*e.g. bumped into a friend, stopped for a coffee*

I went back into the house at: : (specify time)

**Day 4**

Time of day: Morning / Lunch / Afternoon / Evening (please circle)

I went out of the house at: : (specify time)

I was (tick all that apply) : ❒ Alone	❒ With my partner	❒ With a relative	❒ With a friend	❒ Walking a dog	❒ Other (please specify):

I went to (specify the location):

This location is:

❒ Outside my home (e.g. porch, garage, garden)

❒ Within my local neighbourhood (less than ½ mile away)

❒ Outside my local neighbourhood but within town (less than 5 miles)

❒ Outside town (less or more than 10 miles)

The main purpose of going out was:

When you were outside about how long did you spend:

Walking………………………. In a car………………………………………….

Sitting…………………………..Using public transport…………………..

Cycling………………………….Other……………………………………………

While I was out I also did:

*e.g. bumped into a friend, stopped for a coffee*

I went back into the house at: : (specify time)

**Day 4**

Time of day: Morning / Lunch / Afternoon / Evening (please circle)

I went out of the house at: : (specify time)

I was (tick all that apply) : ❒ Alone	❒ With my partner	❒ With a relative	❒ With a friend	❒ Walking a dog	❒ Other (please specify):

I went to (specify the location):

This location is:

❒ Outside my home (e.g. porch, garage, garden)

❒ Within my local neighbourhood (less than ½ mile away)

❒ Outside my local neighbourhood but within town (less than 5 miles)

❒ Outside town (less or more than 10 miles)

The main purpose of going out was:

When you were outside about how long did you spend:

Walking………………………. In a car………………………………………….

Sitting…………………………..Using public transport…………………..

Cycling………………………….Other……………………………………………

While I was out I also did:

*e.g. bumped into a friend, stopped for a coffee*

I went back into the house at: : (specify time)

**Day 4**

Time of day: Morning / Lunch / Afternoon / Evening (please circle)

I went out of the house at: : (specify time)

I was (tick all that apply) : ❒ Alone	❒ With my partner	❒ With a relative	❒ With a friend	❒ Walking a dog	❒ Other (please specify):

I went to (specify the location):

This location is:

❒ Outside my home (e.g. porch, garage, garden)

❒ Within my local neighbourhood (less than ½ mile away)

❒ Outside my local neighbourhood but within town (less than 5 miles)

❒ Outside town (less or more than 10 miles)

The main purpose of going out was:

When you were outside about how long did you spend:

Walking………………………. In a car………………………………………….

Sitting…………………………..Using public transport…………………..

Cycling………………………….Other……………………………………………

While I was out I also did:

*e.g. bumped into a friend, stopped for a coffee*

I went back into the house at: : (specify time)

**Day 4**

Time of day: Morning / Lunch / Afternoon / Evening (please circle)

I went out of the house at: : (specify time)

I was (tick all that apply) : ❒ Alone	❒ With my partner	❒ With a relative	❒ With a friend	❒ Walking a dog	❒ Other (please specify):

I went to (specify the location):

This location is:

❒ Outside my home (e.g. porch, garage, garden)

❒ Within my local neighbourhood (less than ½ mile away)

❒ Outside my local neighbourhood but within town (less than 5 miles)

❒ Outside town (less or more than 10 miles)

The main purpose of going out was:

When you were outside about how long did you spend:

Walking………………………. In a car………………………………………….

Sitting…………………………..Using public transport…………………..

Cycling………………………….Other……………………………………………

While I was out I also did:

*e.g. bumped into a friend, stopped for a coffee*

I went back into the house at: : (specify time)

**Day 5**

Time of day: Morning / Lunch / Afternoon / Evening (please circle)

I went out of the house at: : (specify time)

I was (tick all that apply) : ❒ Alone	❒ With my partner	❒ With a relative	❒ With a friend	❒ Walking a dog	❒ Other (please specify):

I went to (specify the location):

This location is:

❒ Outside my home (e.g. porch, garage, garden)

❒ Within my local neighbourhood (less than ½ mile away)

❒ Outside my local neighbourhood but within town (less than 5 miles)

❒ Outside town (less or more than 10 miles)

The main purpose of going out was:

When you were outside about how long did you spend:

Walking………………………. In a car………………………………………….

Sitting…………………………..Using public transport…………………..

Cycling………………………….Other……………………………………………

While I was out I also did:

*e.g. bumped into a friend, stopped for a coffee*

I went back into the house at: : (specify time)

**Day 5**

Time of day: Morning / Lunch / Afternoon / Evening (please circle)

I went out of the house at: : (specify time)

I was (tick all that apply) : ❒ Alone	❒ With my partner	❒ With a relative	❒ With a friend	❒ Walking a dog	❒ Other (please specify):

I went to (specify the location):

This location is:

❒ Outside my home (e.g. porch, garage, garden)

❒ Within my local neighbourhood (less than ½ mile away)

❒ Outside my local neighbourhood but within town (less than 5 miles)

❒ Outside town (less or more than 10 miles)

The main purpose of going out was:

When you were outside about how long did you spend:

Walking………………………. In a car………………………………………….

Sitting…………………………..Using public transport…………………..

Cycling………………………….Other……………………………………………

While I was out I also did:

*e.g. bumped into a friend, stopped for a coffee*

I went back into the house at: : (specify time)

**Day 5**

Time of day: Morning / Lunch / Afternoon / Evening (please circle)

I went out of the house at: : (specify time)

I was (tick all that apply) : ❒ Alone	❒ With my partner	❒ With a relative	❒ With a friend	❒ Walking a dog	❒ Other (please specify):

I went to (specify the location):

This location is:

❒ Outside my home (e.g. porch, garage, garden)

❒ Within my local neighbourhood (less than ½ mile away)

❒ Outside my local neighbourhood but within town (less than 5 miles)

❒ Outside town (less or more than 10 miles)

The main purpose of going out was:

When you were outside about how long did you spend:

Walking………………………. In a car………………………………………….

Sitting…………………………..Using public transport…………………..

Cycling………………………….Other……………………………………………

While I was out I also did:

*e.g. bumped into a friend, stopped for a coffee*

I went back into the house at: : (specify time)

**Day 5**

Time of day: Morning / Lunch / Afternoon / Evening (please circle)

I went out of the house at: : (specify time)

I was (tick all that apply) : ❒ Alone	❒ With my partner	❒ With a relative	❒ With a friend	❒ Walking a dog	❒ Other (please specify):

I went to (specify the location):

This location is:

❒ Outside my home (e.g. porch, garage, garden)

❒ Within my local neighbourhood (less than ½ mile away)

❒ Outside my local neighbourhood but within town (less than 5 miles)

❒ Outside town (less or more than 10 miles)

The main purpose of going out was:

When you were outside about how long did you spend:

Walking………………………. In a car………………………………………….

Sitting…………………………..Using public transport…………………..

Cycling………………………….Other……………………………………………

While I was out I also did:

*e.g. bumped into a friend, stopped for a coffee*

I went back into the house at: : (specify time)

**Day 6**

Time of day: Morning / Lunch / Afternoon / Evening (please circle)

I went out of the house at: : (specify time)

I was (tick all that apply) : ❒ Alone	❒ With my partner	❒ With a relative	❒ With a friend	❒ Walking a dog	❒ Other (please specify):

I went to (specify the location):

This location is:

❒ Outside my home (e.g. porch, garage, garden)

❒ Within my local neighbourhood (less than ½ mile away)

❒ Outside my local neighbourhood but within town (less than 5 miles)

❒ Outside town (less or more than 10 miles)

The main purpose of going out was:

When you were outside about how long did you spend:

Walking………………………. In a car………………………………………….

Sitting…………………………..Using public transport…………………..

Cycling………………………….Other……………………………………………

While I was out I also did:

*e.g. bumped into a friend, stopped for a coffee*

I went back into the house at: : (specify time)

**Day 6**

Time of day: Morning / Lunch / Afternoon / Evening (please circle)

I went out of the house at: : (specify time)

I was (tick all that apply) : ❒ Alone	❒ With my partner	❒ With a relative	❒ With a friend	❒ Walking a dog	❒ Other (please specify):

I went to (specify the location):

This location is:

❒ Outside my home (e.g. porch, garage, garden)

❒ Within my local neighbourhood (less than ½ mile away)

❒ Outside my local neighbourhood but within town (less than 5 miles)

❒ Outside town (less or more than 10 miles)

The main purpose of going out was:

When you were outside about how long did you spend:

Walking………………………. In a car………………………………………….

Sitting…………………………..Using public transport…………………..

Cycling………………………….Other……………………………………………

While I was out I also did:

*e.g. bumped into a friend, stopped for a coffee*

I went back into the house at: : (specify time)

**Day 6**

Time of day: Morning / Lunch / Afternoon / Evening (please circle)

I went out of the house at: : (specify time)

I was (tick all that apply) : ❒ Alone	❒ With my partner	❒ With a relative	❒ With a friend	❒ Walking a dog	❒ Other (please specify):

I went to (specify the location):

This location is:

❒ Outside my home (e.g. porch, garage, garden)

❒ Within my local neighbourhood (less than ½ mile away)

❒ Outside my local neighbourhood but within town (less than 5 miles)

❒ Outside town (less or more than 10 miles)

The main purpose of going out was:

When you were outside about how long did you spend:

Walking………………………. In a car………………………………………….

Sitting…………………………..Using public transport…………………..

Cycling………………………….Other……………………………………………

While I was out I also did:

*e.g. bumped into a friend, stopped for a coffee*

I went back into the house at: : (specify time)

**Day 6**

Time of day: Morning / Lunch / Afternoon / Evening (please circle)

I went out of the house at: : (specify time)

I was (tick all that apply) : ❒ Alone	❒ With my partner	❒ With a relative	❒ With a friend	❒ Walking a dog	❒ Other (please specify):

I went to (specify the location):

This location is:

❒ Outside my home (e.g. porch, garage, garden)

❒ Within my local neighbourhood (less than ½ mile away)

❒ Outside my local neighbourhood but within town (less than 5 miles)

❒ Outside town (less or more than 10 miles)

The main purpose of going out was:

When you were outside about how long did you spend:

Walking………………………. In a car………………………………………….

Sitting…………………………..Using public transport…………………..

Cycling………………………….Other……………………………………………

While I was out I also did:

*e.g. bumped into a friend, stopped for a coffee*

I went back into the house at: : (specify time)

**Day 7**

Time of day: Morning / Lunch / Afternoon / Evening (please circle)

I went out of the house at: : (specify time)

I was (tick all that apply) : ❒ Alone	❒ With my partner	❒ With a relative	❒ With a friend	❒ Walking a dog	❒ Other (please specify):

I went to (specify the location):

This location is:

❒ Outside my home (e.g. porch, garage, garden)

❒ Within my local neighbourhood (less than ½ mile away)

❒ Outside my local neighbourhood but within town (less than 5 miles)

❒ Outside town (less or more than 10 miles)

The main purpose of going out was:

When you were outside about how long did you spend:

Walking………………………. In a car………………………………………….

Sitting…………………………..Using public transport…………………..

Cycling………………………….Other……………………………………………

While I was out I also did:

*e.g. bumped into a friend, stopped for a coffee*

I went back into the house at: : (specify time)

**Day 7**

Time of day: Morning / Lunch / Afternoon / Evening (please circle)

I went out of the house at: : (specify time)

I was (tick all that apply) : ❒ Alone	❒ With my partner	❒ With a relative	❒ With a friend	❒ Walking a dog	❒ Other (please specify):

I went to (specify the location):

This location is:

❒ Outside my home (e.g. porch, garage, garden)

❒ Within my local neighbourhood (less than ½ mile away)

❒ Outside my local neighbourhood but within town (less than 5 miles)

❒ Outside town (less or more than 10 miles)

The main purpose of going out was:

When you were outside about how long did you spend:

Walking………………………. In a car………………………………………….

Sitting…………………………..Using public transport…………………..

Cycling………………………….Other……………………………………………

While I was out I also did:

*e.g. bumped into a friend, stopped for a coffee*

I went back into the house at: : (specify time)

**Day 7**

Time of day: Morning / Lunch / Afternoon / Evening (please circle)

I went out of the house at: : (specify time)

I was (tick all that apply) : ❒ Alone	❒ With my partner	❒ With a relative	❒ With a friend	❒ Walking a dog	❒ Other (please specify):

I went to (specify the location):

This location is:

❒ Outside my home (e.g. porch, garage, garden)

❒ Within my local neighbourhood (less than ½ mile away)

❒ Outside my local neighbourhood but within town (less than 5 miles)

❒ Outside town (less or more than 10 miles)

The main purpose of going out was:

When you were outside about how long did you spend:

Walking………………………. In a car………………………………………….

Sitting…………………………..Using public transport…………………..

Cycling………………………….Other……………………………………………

While I was out I also did:

*e.g. bumped into a friend, stopped for a coffee*

I went back into the house at: : (specify time)

**Day 7**

Time of day: Morning / Lunch / Afternoon / Evening (please circle)

I went out of the house at: : (specify time)

I was (tick all that apply) : ❒ Alone	❒ With my partner	❒ With a relative	❒ With a friend	❒ Walking a dog	❒ Other (please specify):

I went to (specify the location):

This location is:

❒ Outside my home (e.g. porch, garage, garden)

❒ Within my local neighbourhood (less than ½ mile away)

❒ Outside my local neighbourhood but within town (less than 5 miles)

❒ Outside town (less or more than 10 miles)

The main purpose of going out was:

When you were outside about how long did you spend:

Walking………………………. In a car………………………………………….

Sitting…………………………..Using public transport…………………..

Cycling………………………….Other……………………………………………

While I was out I also did:

*e.g. bumped into a friend, stopped for a coffee*

I went back into the house at: : (specify time)

**Thank you for completing your activity diary, now please answer the following questions:**

1. How have you been feeling this week? (Please note anything you feel is important, whether about physical or mental health, general mood etc)
2. Is there anything you would have liked to have done, or places you would liked to have gone to, but were unable to?

If so, can you tell us what the activity was and why you were unable to do it?

3. Since we saw you last time, has anything happened to you or your family which stands out as important?

This might be changes to your home environment, individual or family circumstances, or other things that have been of interest or concern. Just whatever comes to mind as important since we last saw you.
